# Small RNAs derived from avocado sunblotch viroid and their association with bleaching symptoms: implications for pathogenesis in avocado sunblotch disease

**DOI:** 10.1007/s00705-025-06360-z

**Published:** 2025-09-11

**Authors:** Melissa Joubert, Noëlani van den Berg, Jacques Theron, Velushka Swart

**Affiliations:** 1 Department of Biochemistry, Genetics and Microbiology, Faculty of Natural and Agricultural Sciences, University of Pretoria, Pretoria, Gauteng, South Africa; 2 Hans Merensky Chair in Avocado Research, Forestry and Agricultural Biotechnology Institute, University of Pretoria, Pretoria, Gauteng, South Africa

## Abstract

Avocado sunblotch viroid (ASBVd) is a structured RNA molecule responsible for sunblotch disease of avocado, characterised by distinct chloroses of fruit, leaves, and stems. Despite its impact on avocado, the mechanism by which ASBVd elicits sunblotch symptoms remains unknown. Previous studies on other avsunviroids have shown that viroid-derived small RNAs (vd-sRNAs) with specific sequence mutations can trigger leaf chlorosis via RNA silencing of host genes. Building on this knowledge, we aimed to shed light on the molecular basis of ASBVd pathogenesis by analysing ASBVd sequence variants and ASBVd-sRNAs from bleached and asymptomatic leaf tissues of sunblotch-affected avocado trees. Sequencing of ASBVd clones revealed that variants carrying the pathogenic determinant for bleaching were present in both green and yellow leaf tissues. Next-generation sequencing (NGS) identified ASBVd-sRNAs that varied in abundance between symptomatic and asymptomatic leaf tissues, correlating with viroid titre. We discovered 64 vd-sRNAs spanning the pathogenic region of the ASBVd genome, which were almost exclusively found in yellow tissues. The ASBVd-sRNAs containing the bleaching-associated mutation were predicted to target numerous avocado transcripts for degradation, with 25 of these transcripts significantly downregulated in bleached tissues. Notably, one of these genes, encoding a chloroplastic protein, demonstrated strong evidence of ASBVd-sRNA-guided RNA silencing, presenting a promising candidate for future research into the molecular trigger for ASBVd-induced bleaching symptoms. This study is the first to investigate ASBVd-sRNAs in bleached leaves using NGS. Our findings support the role of RNA silencing in sunblotch symptom development and reveal a unique silencing trigger compared to other avsunviroids.

**Supplementary Information:**

The online version contains supplementary material available at 10.1007/s00705-025-06360-z.

## Introduction

Viroids are the smallest known plant pathogens, existing as single-stranded circular RNA genomes ranging from 246–434 nucleotides (nt) in length, which are not known to code for proteins. To date, over 40 viroid species have been found and classified into two families. The larger of these, the family *Pospiviroidae*, includes viroids that possess central conserved regions (CCRs) within their genomes, replicating in nuclei of host cells by an asymmetrical rolling-circle strategy [1]. The family *Avsunviroidae* has only five known members, all of which replicate in host plastids through a symmetrical rolling-circle strategy, and their self-cleavage is enabled by the formation of hammerhead ribozyme structures during replication [2, 3]. Despite the differences in replication mechanisms between the two families, all viroids depend on host cell RNA polymerases for replication [4]. This error-prone replication process generates mutant genomes with slight sequence variations that accumulate as populations of sequence variants, known as quasispecies [3, 5, 6].

In addition to the differences in their biology, replication, and host cell localisation, members of the two viroid families also differ in the symptoms they cause and their mechanism of pathogenesis (extensively reviewed by Flores et al. [7]). Avsunviroids typically induce specific, localised symptoms in affected hosts, where particular types of chlorosis are restricted to only some parts of the infected plants. These chloroses are directly linked to the downregulation of certain host genes encoding proteins that function in chloroplasts [8–10]. In contrast, members of the family *Pospiviroidae* typically induce systemic symptoms (such as stunting) affecting the entire plant; these symptoms are unlikely to be the direct result of silencing of specific host genes [7].

The downregulation of genes linked to chlorotic phenotypes in avsunviroid-infected plants is driven by post-transcriptional gene silencing (PTGS), mediated by viroid-derived small RNAs (vd-sRNAs) [8–10]. PTGS is a key regulator of plant gene expression, where RNA silencing directs non-coding small RNAs (sRNAs) (20–30 nt) to suppress the translation or accumulation of specific host transcripts [11, 12]. PTGS is typically triggered when double-stranded RNAs, or single-stranded RNAs folded into defined secondary structures, are cleaved by Dicer-like (DCL) RNases into small interfering RNAs (siRNAs) 21, 22, or 24 nt in length. These siRNAs are subsequently bound by plant Argonaute (AGO) proteins to form the RNA-induced silencing complex (RISC), guided by incorporated siRNAs to cleave target messenger RNA (mRNA) or repress its translation, thereby inhibiting expression of the target host gene. Additionally, the RNA silencing machinery serves as a plant defence mechanism against invading pathogens by directing the cleavage of invasive exogenous RNAs, such as those belonging to viral genomes [11–14].

Several studies have shown that viroids from both families are cleaved by the DCLs of the host RNA silencing machinery to produce vd-sRNAs [15–24]. The presence of these vd-sRNAs suggests that their binding to AGOs could lead to RISC-mediated downregulation of host genes, potentially contributing to viroid pathogenesis [13, 25–31]. While the role of RNA silencing in pathogenesis of pospiviroids remains unclear, and is likely not responsible for the initial molecular alteration causing disease symptoms, the role of PTGS in triggering chlorotic symptoms during avsunviroid infection has been well established in three specific interactions [7]. The cause of distinct types of leaf chlorosis in peach infected with peach latent mosaic viroid (PLMVd, genus *Pelamoviroid*), namely, peach calico and peach yellow mosaic, has been investigated in separate studies, revealing that each chlorotic phenotype was triggered by PLMVd sequence variants carrying a specific mutation. In both cases, PLMVd genomes with the distinctive mutations (i.e., the pathogenic determinants) accumulated preferentially in yellow sectors of symptomatic leaves, but not in adjacent green sectors or in asymptomatic leaves. PLMVd-sRNAs containing the pathogenic determinants targeted chloroplastic host mRNAs for degradation by RISC-mediated silencing, resulting in chlorotic phenotypes [8, 9]. The same mechanism of pathogenesis was discovered for chrysanthemum chlorotic mottle viroid (CChMVd; genus *Pelamoviroid*), where a vd-sRNA carrying the mutation associated with chlorotic tissues targeted the mRNA of a chloroplastic transketolase for cleavage, thereby triggering leaf chlorosis [10]. The causal relationships identified in the three interactions, where vd-sRNA-mediated PTGS directly downregulated host genes encoding chloroplast-localised proteins, provide strong evidence that RNA silencing contributes to the “initial molecular lesion” in symptomatic avsunviroid infections [7].

While the mechanisms of pathogenesis for the avsunviroids PLMVd and CChMVd are now better defined, the disease caused by the type member of the family *Avsunviroidae*, avocado sunblotch viroid (ASBVd; genus *Avsunviroid*), remains poorly understood. ASBVd causes avocado sunblotch disease, characterised by the appearance of distinct chlorotic symptoms on infected avocado trees, similar to those observed in PLMVd-infected peach trees. Symptoms of sunblotch disease include the formation of coloured, sunken lesions on avocado fruit, yellow streaks on stems, and chlorosis of leaves [32]. Leaf chlorosis caused by ASBVd has two distinct forms: bleaching, where chlorotic (usually yellow) lesions are separated from adjacent green tissues by distinct margins, and variegation, where leaves have a mosaic-like pattern of chlorosis, with no distinct separation of green and yellow tissues [33].

The association of certain leaf phenotypes with the accumulation of particular ASBVd sequence variants was first investigated in 1994, when viroid genomes from bleached, variegated, and symptomless carrier (fully green) leaves were sequenced, leading to the proposal that the ASBVd-B, ASBVd-V, and ASBVd-SC variants were associated with the respective leaf phenotypes. The study found that the most notable sequence differences involved exchanges or additions of uracil residues within the region 115–118, or of adenine residues within the region 122–128 of the viroid genomes, with these regions together forming the right terminal loop (RTL) of the rod-shaped ASBVd secondary structure [33]. A subsequent study comparing ASBVd variant populations in leaves from asymptomatic avocado trees to those in bleached leaves from a tree with chlorotic symptoms revealed that 21 out of 23 sequenced clones from the bleached ASBVd-infected leaves contained one or two extra uracil residues inserted at positions 115–118 within the viroid genome’s RTL. This finding supports the role of these uracil insertions as the pathogenic determinant for bleaching in ASBVd infections [34, 35]. This proposed pathogenic determinant has, however, not been independently validated, and there is no information regarding the accumulation of certain ASBVd variants in specific leaf tissues (i.e., yellow vs. green) from symptomatic avocado trees.

The involvement of RNA silencing in ASBVd pathogenesis was first suggested after vd-sRNAs were detected in chlorotic ASBVd-infected tissues [16]. Initial studies of asymptomatic tissues from symptomless carrier plants failed to detect the presence of ASBVd-derived sRNAs (ASBVd-sRNAs) [20]. However, a subsequent study found them in the chlorotic portions of symptomatic avocado leaves and fruit, although they were absent in the green portions of the same tissues [16]. Due to the hybridisation methods used in the study, the sequence and size distribution of ASBVd-sRNAs in bleached tissues remain unidentified. Nevertheless, the presence of these vd-sRNAs supports a potential role for PTGS in triggering the bleaching seen in symptomatic ASBVd infections [16], which can now be investigated further using next-generation sequencing (NGS) technologies.

In this study, we aimed to deepen the understanding of ASBVd pathogenesis by investigating the bleaching symptoms triggered by viroid infection of avocado. We analysed the ASBVd variant populations within specific leaf tissues of symptomatic trees and discovered that sequence variants containing the bleaching-associated pathogenic determinant were present in both green (asymptomatic) and yellow (chlorotic) leaf tissues. However, these variants exhibited significant differences in their accumulation levels between different tissues. Using NGS, we characterised ASBVd-sRNAs in these tissues. While substantial variations in the accumulation levels were observed between yellow and green tissues, other characteristics, such as size distribution, polarity, and mapping hotspots, remained consistent. To investigate potential molecular mechanisms, we predicted avocado transcripts that might be degraded by ASBVd-sRNAs containing the pathogenic determinant. Subsequent analysis of putative host target genes revealed downregulated genes that could be associated with the bleaching symptoms observed in avocado sunblotch disease.

## Materials and methods

### Plant material

Four mature, actively-growing avocado trees displaying typical avocado sunblotch disease-associated chlorosis (leaf bleaching and variegation) were identified in two commercial orchards in Tzaneen, Limpopo Province, South Africa, in December 2023. For each tree (treated as separate biological replicates), all fully emerged symptomatic (bleached) leaves were collected, along with five asymptomatic (fully green) leaves from representative branches (Supplementary Table S1). Leaves were kept at 4°C during transport and then flash-frozen in liquid nitrogen. For each biological replicate, yellow sectors of bleached leaves were separated from adjacent green sectors, and these two sample types were separately pooled across leaves for each replicate, forming the SY (yellow) and SG (green) samples, respectively. Additionally, asymptomatic leaves were pooled to form the AS (asymptomatic) samples. Pooled leaf material was ground together for each of the 12 individual samples (four biological replicates for each of the three sample types – SY, SG, and AS) using an IKA^®^ Tube Mill (IKA^®^, Staufen, Germany). A portion of the ground material was sent to Macrogen Genome Center (Seoul, Republic of Korea) for NGS (sRNA and mRNA sequencing), while the remaining material was stored at -70°C for subsequent ASBVd detection and variant sequencing.

### ASBVd detection

RNA was extracted from 50 mg of the ground material for each pooled sample using a modified CTAB method [36]. RNA concentration and quality were assessed using a NanoDrop™ 2000 spectrophotometer (Thermo Fisher Scientific, Waltham, Massachusetts, USA) and by agarose gel electrophoresis. ASBVd cDNA was synthesised from 400 ng of RNA for each sample, as well as known infected and uninfected controls, using a High-Capacity cDNA Reverse Transcription Kit (Applied Biosystems™, Thermo Fisher Scientific) with modified conditions, using the ASBVd-specific primers SB1-F1 (5’-TGGGAAGAACACTGATGAG-3’) and SB1-R1 (5’-TCTTTCCCTGAAGAGACGA-3’) [33]. These primers were also used for a 15-cycle PCR pre-amplification of the ASBVd cDNA template using FastStart™ Taq DNA Polymerase (Roche Applied Science, Merck KGaA, Darmstadt, Germany) on a Veriti™ 96-Well Fast Thermal Cycler (Applied Biosystems™, Thermo Fisher Scientific). The pre-amplification products served as templates in a nested, semi-quantitative real-time PCR using TaqMan™ Gene Expression Master Mix (Applied Biosystems™, Thermo Fisher Scientific) on a CFX Connect™ Real-Time PCR System (Bio-Rad Laboratories, Hercules, California, USA). Reaction conditions for pre-amplification and real-time PCR have been described previously [37]. ASBVd cDNA synthesis reactions and pre-amplifications were conducted separately for yellow and green tissue samples, and all pre-amplification products were run on a single 96-well plate in the real-time PCR. Positive ASBVd detection was indicated by fluorescence in the TaqMan™ assay, while the absence of fluorescence suggested undetectable or very low viroid levels.

### Sequencing of ASBVd variants

For each sample with detected ASBVd, viroid cDNA (synthesised from 400 ng of RNA) served as the template for PCR amplification using Phusion™ High-Fidelity DNA Polymerase (Thermo Fisher Scientific) as described previously [37]. PCR products were purified from a 1.5% agarose gel using a Nucleospin^®^ Gel and PCR Clean-up Kit (MACHEREY-NAGEL, MACHEREY-NAGEL GmbH & Co. KG, Düren, Germany) as per manufacturer’s instructions. Concentrations were measured as before, and products were cloned into *Escherichia coli* DH5α using an Invitrogen Zero Blunt^®^ TOPO PCR Cloning Kit (Thermo Fisher Scientific). Bacterial colonies were incubated overnight in Luria-Bertani broth supplemented with 50 µg of kanamycin per ml at 37°C with shaking at 150 rpm. Plasmids were extracted using a Nucleospin^®^ Plasmid Kit (MACHEREY-NAGEL), and five plasmids per sample were sequenced in both directions using M13 primers (M13-F, 5’-GTAAAACGACGACGGCCAGT-3’; M13-R, 5’-CAGGAAACAGCTATGAC-3’). BigDye™ v3.1 (Applied Biosystems™, Thermo Fisher Scientific) reactions and sodium acetate precipitation of sequencing products were carried out as described previously [37]. Purified products were sequenced using an ABI 3500xl Genetic Analyser (Applied Biosystems™, Thermo Fisher Scientific) by the DNA Sanger Sequencing Facility at the University of Pretoria (Faculty of Natural and Agricultural Sciences). Raw sequences were analysed and aligned to known variants using CLC Main Workbench v21.0.1 (QIAGEN Bioinformatics, Aarhus, Denmark), and secondary structures of ASBVd variants were predicted using Structure Editor v1.0 of RNAstructure v6.4 [38].

### Small-RNA sequencing and analysis

For NGS, total RNA was extracted from all samples by Macrogen, with RNA concentration and quality assessed using an Agilent RNA 6000 Nano chip on a 2100 Bioanalyzer (Agilent Technologies, Santa Clara, California, USA). Library construction for sRNA sequencing was performed by Macrogen using a SMARTer^®^ smRNA-Seq Kit for Illumina^®^ (Takara Bio USA, Inc., San Jose, California, USA), and libraries were sequenced (2 × 100 bp paired-end) on an Illumina Novaseq 6000 platform (Illumina, Inc., San Diego, California, USA). Raw sRNA reads were trimmed with Cutadapt v4.8 [39] to remove adapter sequences, subsequently retaining only 18- to 26-nt reads. The quality of the filtered reads was assessed using FastQC v0.11.9 [40] and summarised using MultiQC v1.13a [41]. To identify ASBVd-sRNAs, command-line BLAST + v2.15.0 (blastn-short) was used to match reads with 100% sequence identity to known ASBVd variants [33, 37, 42] or those sequenced in this study.

### Target prediction and expression analysis

To identify avocado transcripts that are potentially targeted by ASBVd-sRNAs in PTGS, the psRNATarget web server [43] was used for *in silico* prediction. ASBVd-sRNAs derived from the pathogenic region of the viroid genome were queried against the coding sequences (CDSs) and transcripts of the *Persea americana* West Indian (WI) pure accession genome (Peame105) (Avocado Genome Consortium, personal communication) using default parameters and an E-value cutoff of 3.5. Potential targets were filtered for those downregulated in yellow (SY) samples relative to green (SG and AS) samples. Expression data from mRNA sequencing were used, with libraries prepared for pooled SY, SG, and AS samples from three biological replicates (trees 1, 2, and 3) using a TruSeq^®^ Stranded mRNA Library Prep Kit (Illumina, San Diego, California, USA) and sequenced on an Illumina NovaSeqX platform (2 × 150 bp paired-end) by Macrogen. Sequencing reads were trimmed, quality-checked, aligned to the Peame105 genome using HISAT2 v2.2.1 [44], and quantified using featureCounts [45] as described previously [37]. Analysis of count data with DESeq2 v1.38.3 [46] in RStudio v2024.04.2.764 [47] used the Wald test to determine differential gene expression (DGE) [37]. DGE analysis was performed first between SY and SG samples, and then for SY and SG relative to AS samples. Targets were considered downregulated if the log_2_(fold change) (log_2_FC) was < 0 and the adjusted *p*-value (*p*adj) was ≤ 0.1 in SY samples across both comparisons.

Downregulated genes were further analysed to verify psRNATarget predictions of PTGS targets in avocado. Target prediction was repeated in psRNATarget with stricter parameters, doubling penalties in the seed region (positions 2–13 of the sRNA, reading from the 5’ end) as recommended by Fahlgren and Carrington [48], and the E-value cutoff set to 3.5. Predicted ASBVd-sRNA:mRNA duplexes were assessed using RNAhybrid [49] to identify hybrids with minimum free energy (mfe) < 0 kcal/mol, indicating a higher likelihood of duplex formation. Subcellular localisation of target-encoded proteins was predicted using DeepLoc v2.1 [50], and homologues in *Arabidopsis thaliana* were identified via BLASTp of target amino acid sequences against Araport 11 proteins sequences on TAIR (The Arabidopsis Information Resource; https://www.arabidopsis.org/tools/blast/).

## Results

### The pathogenic determinant associated with bleaching is present in yellow and green leaf tissues of sunblotch-affected avocado trees

**Fig. 1:**
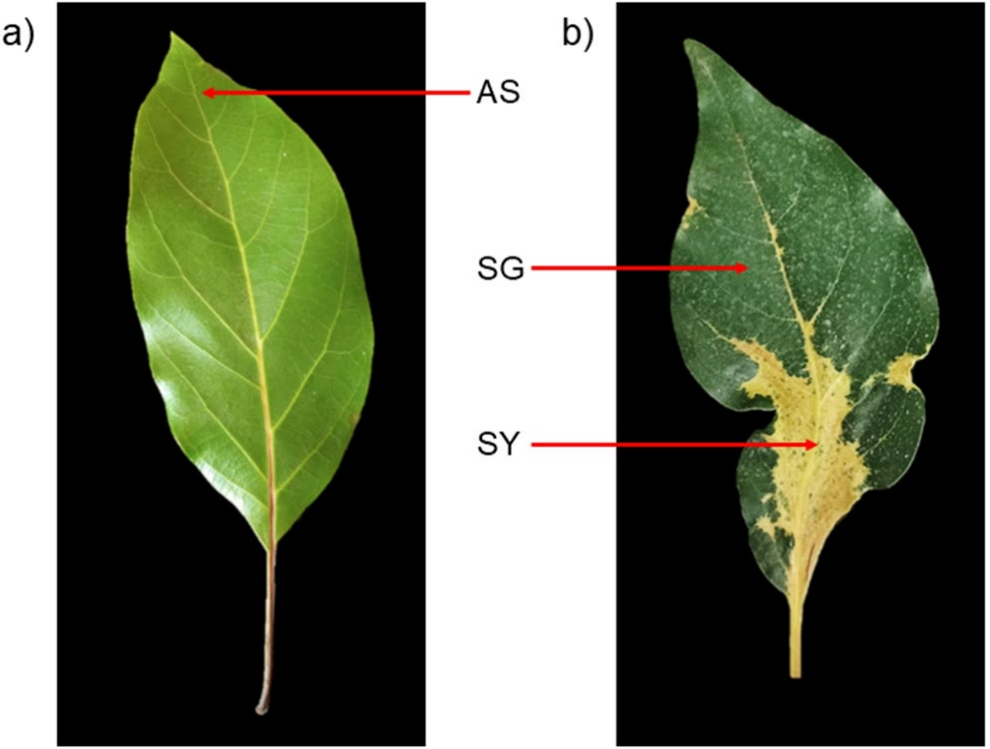
Two leaf phenotypes in sunblotch-affected avocado trees analysed in this study. (**a**) Asymptomatic leaves were entirely green, with no chlorosis, and were representative of most of the leaves on trees infected with avocado sunblotch viroid (ASBVd). (**b**) Bleached leaves displayed chlorotic (usually yellow) lesions with distinct margins separating them from green tissues on the same leaf, appearing sporadically on symptomatic trees. AS samples included pooled green tissue from asymptomatic leaves (**a**), while SG and SY samples comprised pooled green and yellow sectors, respectively, from bleached leaves (**b**)

To confirm the previously identified pathogenic determinant in the ASBVd genome associated with bleaching symptoms in sunblotch-affected avocado trees, we analysed asymptomatic leaves as well as yellow and green tissues of bleached leaves (Fig. 1) from symptomatic trees. Semi-quantitative real-time PCR detected ASBVd in both SY (yellow sector) and SG (green sector) leaf samples for all four trees, indicated by fluorescence (Supplementary Table S2). SY samples consistently showed higher ASBVd titres (lower Ct values) when compared to SG samples from the same symptomatic leaves, which had lower viroid concentrations. AS samples (asymptomatic, entirely green leaves) exhibited overall lower ASBVd titres, with only two samples (tree 2 and tree 4) testing positive. AS samples from trees 1 and 3 were either free of ASBVd or had viroid titres that were too low to be detected in this assay, with results being indistinguishable from uninfected and non-template controls. Positive controls from asymptomatic leaves of known symptomless carrier trees were amplified successfully. Positive amplification results were confirmed by gel electrophoresis of real-time PCR products, which showed single bands (~ 155 bp) in samples and controls that exhibited fluorescence in the TaqMan™ assay. No bands were observed in the two AS samples or negative controls lacking fluorescence (Supplementary Fig. S1).

**Fig. 2:**
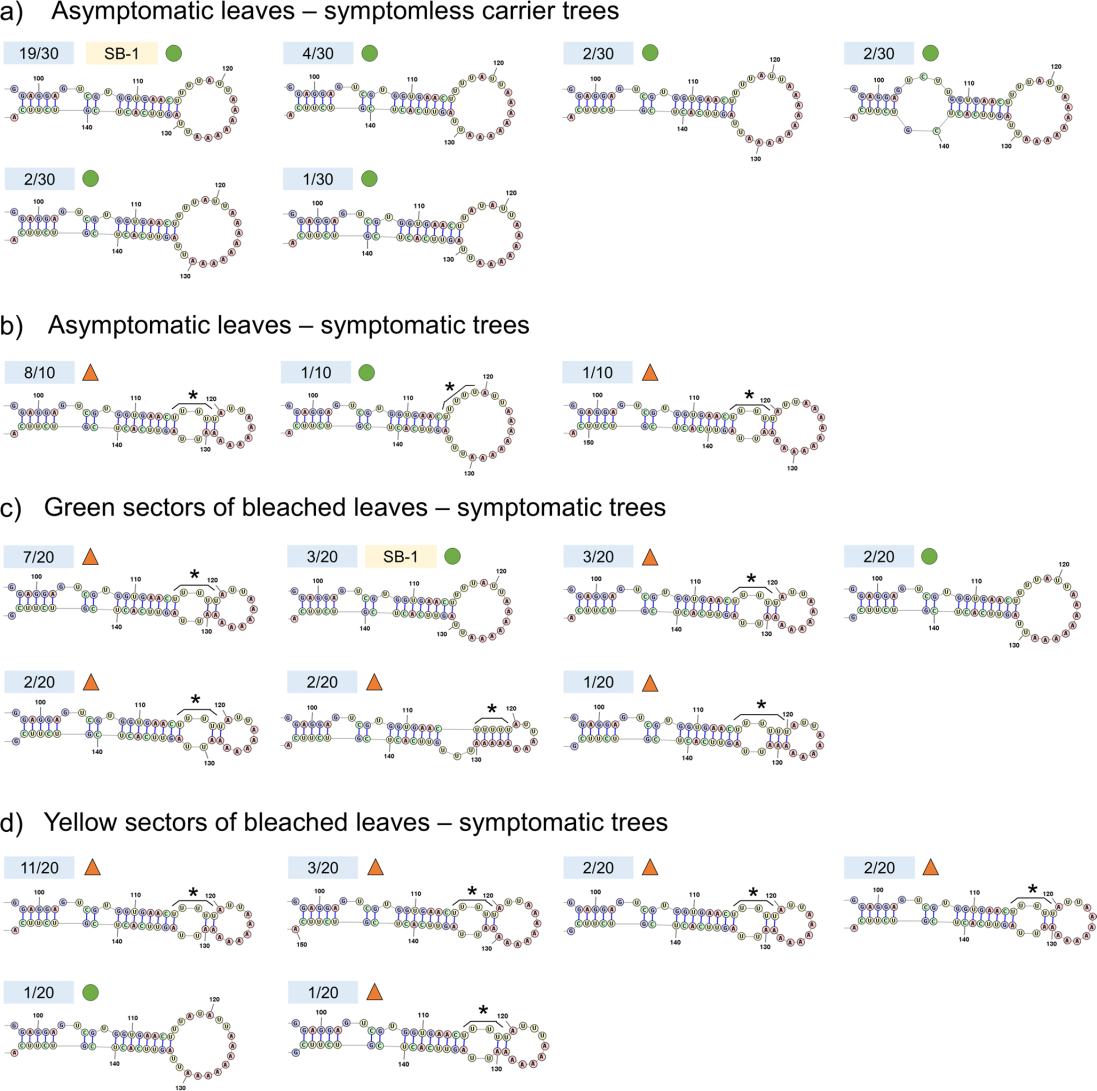
Sequence mutations occurring within the right terminal loop (RTL) of avocado sunblotch viroid (ASBVd). The proportion of clones with specific alterations in the ASBVd RTL structure is indicated for each leaf phenotype across two studies ([37] and this study). RTL structures are shown for clones from (**a**) asymptomatic leaves on symptomless carrier trees, (**b**) asymptomatic leaves on trees with chlorotic symptoms, (**c**) green sectors of bleached leaves, and (**d**) yellow sectors of bleached leaves. The numbers in blue blocks represent the abundance of clones with altered RTL sequences, while "SB-1" in a yellow block indicates an RTL sequence that is identical to that of the ASBVd type strain SB-1. Most variants from symptomatic trees (**b**-**d**) contained an additional uracil residue within bases 115–118 (marked by asterisks above the relevant region) and had RTL structures ending in two small loops: one internal loop and one terminal loop (indicated by orange triangles above the corresponding secondary structures). No clones sequenced from symptomless plants (**a**) had the uracil insertion at position 115–118, and all symptomless carrier variants had RTL secondary structures terminating in a single enlarged hairpin loop (indicated by green circles above the secondary structures)

Five ASBVd cDNA clones were sequenced from each of the 10 samples with successful viroid detection, yielding 50 ASBVd sequences in total (10 from AS, 20 from SG, and 20 from SY). The sequences of these quasispecies populations were compared to the ASBVd type strain SB-1 [42] and the previously reported ASBVd-B and ASBVd-SC variants [33]. To verify the previously reported pathogenic determinant of bleaching, 30 additional ASBVd sequences obtained from asymptomatic nursery trees in a previous study [37] were also included in the alignment. Multiple sequence alignment (MSA) highlighted key differences within region 115–128 relative to SB-1 (Supplementary Fig. S2) – part of the RTL region of the ASBVd secondary structure (Fig. 2).

The number of adenine residues in the RTL (positions 122–128 of SB-1) was highly variable among different isolates, even within sample types, and specific genome alterations pertaining to this run of adenine residues were not found to be associated with individual tissue types (Supplementary Fig. S2). When distinguishing between the different sample types from which variants were obtained, secondary structure prediction revealed structural differences within the RTL of variants isolated from symptomatic trees when compared to those present in symptomless carrier trees (Fig. 2). All ASBVd variants sequenced from asymptomatic leaves of symptomless carrier trees were predicted to have an RTL structure resembling that of SB-1: a hairpin loop composed of an 8-bp stem ending in a single large terminal loop (Fig. 2a). In contrast, secondary structure prediction showed that the RTL of the majority of variants sequenced from green (AS and SG) and yellow (SY) leaf tissues of symptomatic trees contained a compound loop structure in which the 8-bp stem preceded a small internal loop before terminating in a small hairpin loop (Fig. 2b-d).

When examining the primary sequence of the RTL region in variants obtained from different sample types, the most notable alteration was the insertion of at least one additional uracil within the run of uracil residues at positions 115–118 in SB-1 – a mutation that occurred only in samples isolated from symptomatic avocado trees (Supplementary Fig. S2). No variants from green leaves of symptomless carrier trees, including ASBVd-SC [33] and all the SCHassKZN variants sequenced previously [37], contained this additional uracil. This mutation was, however, present in the ASBVd-B variant [33], and in 44 of 50 clones sequenced in this study from both green (SG) and yellow (SY) tissues of symptomatic trees (Supplementary Fig. S2; structures with asterisks in Fig. 2b-d). All 10 AS sequences contained the additional uracil insertion associated with bleaching (Fig. 2b), which was absent in clones from asymptomatic leaves of symptomless carrier trees [37] (Fig. 2a). These findings, taken together with those of previous studies [33–35, 37], indicate that the addition of at least one uracil at positions 115–118 of the ASBVd genome is associated with the chlorotic symptoms in sunblotch-affected trees. This additional uracil was therefore considered the ASBVd bleaching-associated pathogenic determinant in our subsequent analyses.

### ASBVd-sRNAs with the bleaching-associated mutation accumulate primarily in yellow leaf tissues of symptomatic ASBVd-infected avocado trees

To confirm ASBVd cleavage into sRNAs by the host RNA silencing machinery and to compare sRNAs across tissue types, we used NGS to sequence the sRNAs from each of the 12 samples. Illumina sequencing yielded 10.6–17.8 M reads per sample, although most were discarded after filtering by size. On average, 62.7% of the reads were < 18 nt, and 9.5% were > 26 nt (Supplementary Fig. S3a). Size distribution analysis of the remaining ~ 27.8% reads showed a peak at 21 nt in all samples, with many samples also showing high abundance at 23 and 24 nt (Supplementary Fig. S3b).

Filtered sRNA-sequencing reads were mapped to ASBVd sequence variants to identify vd-sRNAs. The four SY replicates contained 25,725–38,000 ASBVd-sRNAs per sample, SG samples had 79–684, and AS samples had 0–57 ASBVd-sRNAs per tree (Supplementary Table S3). No vd-sRNAs were detected in the AS samples that tested negative for the viroid using the TaqMan™ assay (Tree 1_AS and Tree 3_AS). The ASBVd-sRNA counts among sample types were not proportional to the total number of sRNAs per library type, but were instead correlated to the viroid accumulation levels detected in the semi-quantitative real-time PCR assay. Combining reads for each tissue type and normalising to reads per million showed that SY samples contained nearly 100-fold more ASBVd-sRNAs than SG samples, while AS samples contained negligible amounts (Table 1).

**Table 1:** Combined number of ASBVd-sRNA reads in different tissue types sampled from four sunblotch-affected avocado trees

Sample type	Number of raw sRNA reads	Number of reads after filtering^a^	Number of ASBVd-sRNAs in filtered library	ASBVd-sRNAs per million filtered reads
AS	57,437,324	16,402,675	60	3.66
SG	52,882,504	14,452,594	1,324	91.61
SY	52,846,051	14,256,026	122,842	8,616.85

^a^Number of reads remaining after adapter trimming and filtering to maintain only 18–26 nt reads. AS, asymptomatic (entirely green) leaves from symptomatic trees; SG, green sectors of bleached leaves from symptomatic trees; SY, yellow sectors of bleached leaves from symptomatic trees

Despite variations in ASBVd-sRNA abundance across sample types, the polarity ratios (abundance of sRNAs from specific genome strands) and size distribution of these vd-sRNAs were similar between green and yellow tissues (Supplementary Fig. S4). Most of the ASBVd-sRNAs identified in our analysis originated from the antisense (minus; -) strand of the ASBVd genome in all samples (Supplementary Fig. S4a). In SG and SY samples, only 24–26% of ASBVd-sRNAs were from the sense (plus; +) strand, while AS samples had 42% (+) strand ASBVd-sRNAs, likely skewed due to the very small number of vd-sRNAs in AS samples. The size distribution was consistent across the sample types, with most reads being 19–21 nt long, with slightly fewer 22-nt reads (Supplementary Fig. S4b).

**Fig. 3:**
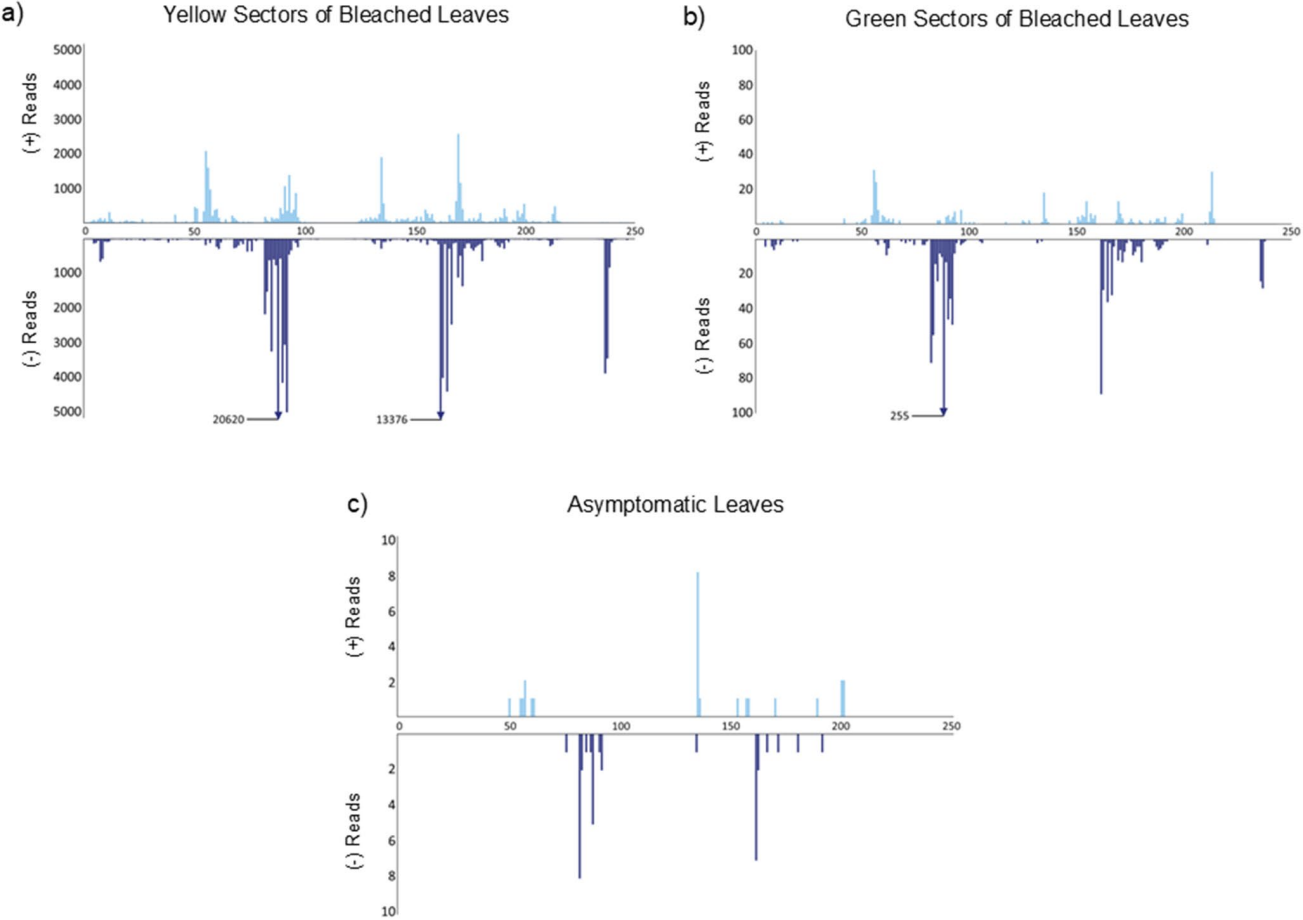
Mapping profiles of avocado sunblotch viroid-derived small RNAs (ASBVd-sRNAs) in avocado leaf tissue types. Hotspot mapping of ASBVd-sRNAs from four biological replicates is shown for (**a**) yellow tissue from bleached leaves, (**b**) green tissue from bleached leaves, and (**c**) green tissue from asymptomatic leaves of symptomatic trees. The *x*-axis shows the locations of the 5’ termini of ASBVd-sRNAs relative to the ASBVd genome, while the *y*-axis indicates the read counts for each mapped position. ASBVd-sRNAs from the sense (+) strand appear on the upper panels, oriented 5’ to 3’ from left to right along the *x*-axis, while antisense (-) strand ASBVd-sRNAs appear on lower panels, oriented 5’ to 3’ from right to left

Mapping of vd-sRNAs to the ASBVd genome showed slight differences in the regions of the viroid from which (+) and (-) ASBVd-sRNAs were generated, with similar profiles for all sample types (Fig. 3). Hotspots for (+) vd-sRNAs appeared at positions 55–57 and 135, while (-) vd-sRNAs were most abundant at positions 82–92, 162–167, and 237–238 of the genome. Notably, the RTL region containing the pathogenic determinant (corresponding to positions 115–118 of SB-1) did not serve as a hotspot for either strand. These mapping hotspots remained consistent across SY, SG, and AS samples, despite significant differences in ASBVd-sRNA abundance.

To identify ASBVd-sRNAs specifically linked to bleaching, we analysed unique reads in SY samples mapping to the RTL of the (+) or (-) strand of the viroid genome. A total of 64 unique ASBVd-sRNAs were found to originate from this pathogenic region, with 52 containing the additional uracil (or complementary adenine in the (-) genome) identified as the pathogenic determinant of ASBVd (Supplementary Table S4). These ASBVd-sRNAs, termed ASBVdB-sRNAs, had a relatively even size distribution of 18–26 nt, with most (40/64) originating from the (+) strand. Only one ASBVdB-sRNA found in SY samples (ASBVdB-sRNA38a) was also found in a single SG sample, while all other ASBVdB-sRNAs were exclusive to yellow tissues. Three additional ASBVd-sRNAs from SG samples originated from the RTL, but none contained the pathogenic determinant associated with bleaching and were therefore excluded from further analysis. No AS samples contained ASBVd-sRNAs mapping to the RTL of the genome.

### ASBVd-sRNAs containing the pathogenic determinant of bleaching are predicted to target avocado mRNAs for downregulation by PTGS

We used psRNATarget to assess the potential of 64 ASBVdB-sRNAs to target host mRNAs for degradation by RISC-mediated silencing. Predictions indicated that most host transcripts were targeted by multiple ASBVdB-sRNAs, and individual ASBVdB-sRNAs mapped to multiple avocado mRNAs. After accounting for redundancy, 460 unique host mRNAs were identified as potential targets of 50 ASBVdB-sRNAs, using an E-value cutoff of 3.5 with default parameters. Given the likelihood of false positives among these targets, we applied DGE analysis to identify avocado mRNAs that were downregulated in yellow tissues.

**Fig. 4:**
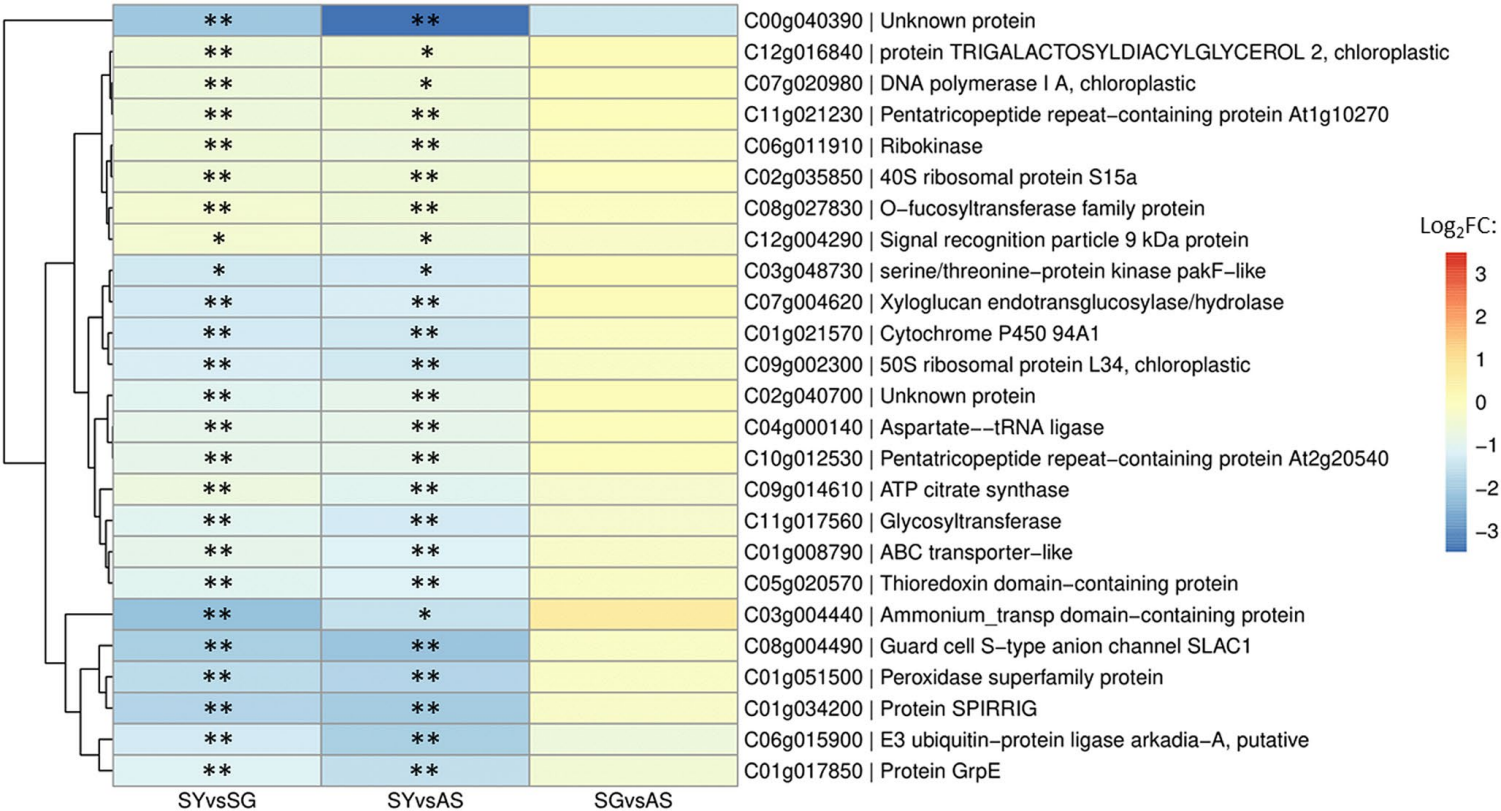
Heat map of the genes significantly downregulated in yellow tissues in ASBVd-infected trees displaying bleaching symptoms. These 25 avocado genes were all predicted to be targeted for RISC-mediated degradation by avocado sunblotch viroid-derived small RNAs (ASBVd-sRNAs) based on *in silico* analysis. Gene names and annotations are from the *Persea americana* West Indian pure accession genome (Peame105). Expression levels, shown as log_2_(fold change) (log_2_FC), are colour-coded for yellow sectors of bleached leaves (SY) relative to green sectors of bleached leaves (SG) (left panel), SY vs. AS (asymptomatic leaves from symptomatic trees) (middle panel), and SG vs. AS samples (right panel). Single asterisks indicate significant downregulation at *p*adj ≤ 0.1, while double asterisks denote significance at *p*adj ≤ 0.05. Genes with similar expression patterns are clustered together according to the dendrogram to the left of the heat map

Global transcriptome data for SY, SG, and AS tissue samples (manuscript in preparation) were used to analyse downregulated genes (log_2_FC < 0) in yellow tissues compared to green tissues. When SY expression was normalised against SG, 2,578 genes were significantly downregulated at *p*adj < 0.1, 2,008 of which were significant at *p*adj < 0.05. Using AS as the baseline expression, 3,206 genes were significantly downregulated in SY samples at *p*adj < 0.1 (2,491 at *p*adj < 0.05), while only eight genes were significantly downregulated in SG at *p*adj < 0.1 (five at *p*adj < 0.05). Among the 460 predicted host targets, only 44 (~ 10%) were significantly downregulated (at *p*adj ≤ 0.1) in yellow tissues in at least one dataset (SY vs. SG or SY vs. AS). To identify candidates most likely to be silenced by RNA-mediated mechanisms in bleached tissues, we focused on genes significantly downregulated in SY samples compared to both SG and AS (*p*adj ≤ 0.1). This yielded 25 candidate target genes (Fig. 4; Supplementary Fig. S5), 20 of which were significantly downregulated at *p*adj ≤ 0.05 in both datasets. None of these genes were significantly differentially expressed in SG samples compared to AS samples.

We further analysed the duplexes formed between ASBVdB-sRNAs and the 25 avocado transcripts downregulated in yellow tissues to identify the genes most likely to be silenced by PTGS (Supplementary Table S5). Using a stricter scoring matrix in psRNATarget as recommended by Fahlgren and Carrington [48], only 13 transcripts formed duplexes with ASBVdB-sRNAs at an E-value threshold of 3.5 (shown to be sensitive and specific in arabidopsis [48]). RNAhybrid analysis revealed that the 12 host transcripts excluded by stricter psRNATarget criteria had mfe ≥ 0 kcal/mol, supporting their classification as false predictions. Of the 13 remaining candidate mRNA targets, two were excluded, as RNAhybrid did not predict duplex formation, and four others lacked the pathogenic determinant within the ASBVdB-sRNA:mRNA target duplexes. The final analysis focused on seven avocado mRNAs targeted by 27 ASBVdB-sRNAs, ranging in size from 19 to 25 nt (indicated by “*” in Supplementary Table S4).

We subsequently focused only on 21- to 22-nt ASBVdB-sRNAs, the sizes required for AGO1 loading and RISC-guided mRNA cleavage [26, 51, 52]. Filtering duplex predictions by sRNA size yielded four avocado genes as candidates for RISC-mediated downregulation by bleaching-associated 21- and 22-nt ASBVdB-sRNAs. These included *C01g021570* (*cytochrome P450 94A1*; *CYP94A1*), *C11g017560* (a glycosyltransferase), *C12g016840* (*TRIGALACTOSYLDIACYLGLYCEROL2*; *TGD2*), and *C09g002300* (*plasmid ribosomal protein 34 of the 50S subunit*; *PRPL34*). These genes were targeted by multiple ASBVdB-sRNAs meeting scoring criteria (Table 2). E-value scores, mfe values, and pathogenic determinant locations suggested that their downregulation in yellow tissues may be the result of RISC-mediated targeting by bleaching-associated ASBVdB-sRNAs.

**Table 2:**
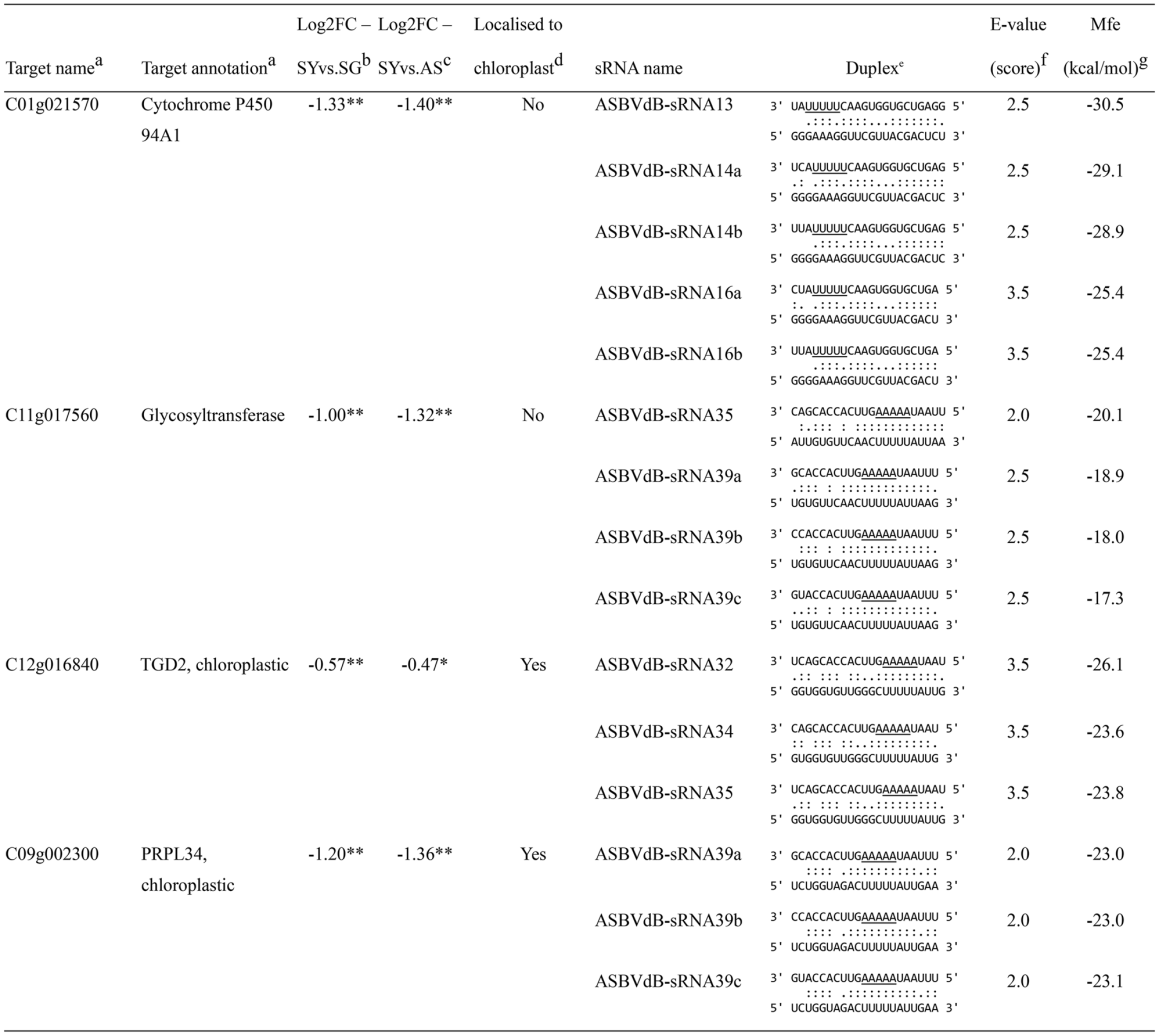
Avocado transcripts predicted to be targeted for degradation by ASBVdB-sRNAs containing the bleaching-associated mutation

^a^Target name and putative annotation as identified in the *Persea americana* West Indian pure accession genome (Peame105). TGD2: Protein TRIGALACTOSYLDIACYLGLYCEROL 2; PRPL34: 50S ribosomal subunit protein L34

^b^Log_2_(fold change) (log_2_FC) of target gene expression in yellow sectors (SY) relative to green sectors (SG) of bleached leaves

^c^Log_2_FC of target gene expression in yellow sectors (SY) of bleached leaves relative to asymptomatic leaves (AS) from symptomatic trees

^d^Protein localisation to chloroplasts as predicted by DeepLoc 2.1 (https://services.healthtech.dtu.dk/services/DeepLoc-2.1)

^e^Duplexes predicted to form between mRNA targets and ASBVdB-sRNAs by psRNATarget (https://www.zhaolab.org/psRNATarget). ASBVdB-sRNAs are at the top of each duplex (3’-5’), with the region of the sRNA containing the pathogenic determinant underlined, and target sequences of avocado mRNAs are at the bottom (5’-3’). Perfect base-pairing is shown by “:”, G:U base-pairing is indicated by “.”, and mismatches or bulges are represented by open spaces in each duplex

^f^The E-value (score) of each target:ASBVd-sRNA duplex as calculated by psRNATarget, using the scoring matrix designed by Fahlgren & Carrington [48]

^g^Minimum free energy (mfe) of the target:ASBVd-sRNA duplex as predicted by RNAhybrid (https://bibiserv.cebitec.uni-bielefeld.de/rnahybrid)

*Expression of target is significant at adjusted *p*-value (*p*adj) ≤ 0.1

**Expression of target is significant at *p*adj ≤ 0.05

To determine whether downregulation of any of the four shortlisted host targets could be directly linked to bleaching symptoms, we investigated the subcellular localisation of their encoded proteins and identified functionally characterised homologues in arabidopsis. Among the avocado targets, CYP94A1 and the glycosyltransferase were not localised to chloroplasts in host cells. Furthermore, the characterisation of their arabidopsis homologues indicated that the encoded avocado proteins are involved in enzymatic reactions within host cells. Consequently, the downregulation of these targets is unlikely to be directly associated with the onset of bleaching symptoms in ASBVd-infected trees.

The remaining two avocado targets, *TGD2* and *PRPL34*, represented nuclear-encoded genes whose proteins were predicted to localise to chloroplasts in host cells, making them strong candidates for the initial triggers of bleaching symptoms. Functional disruption of chloroplastic proteins could directly result in chlorotic phenotypes. The *P. americana* TGD2 shared 70% sequence identity with its arabidopsis homologue (AT3G20320.1), a chloroplastic lipid transporter that is important for thylakoid biogenesis [53]. Avocado PRPL34 exhibited 75% sequence identity to its arabidopsis homologue (AT1G29070.1), which Gene Ontology predicts to be involved in the translation of chloroplastic transcripts. The proposed functions of these chloroplastic avocado proteins suggests a link between their reduced transcript levels and the chlorotic symptoms observed in bleached leaves. Notably, *PRPL34* had stronger evidence for its downregulation being due to degradation mediated by ASBVdB-sRNAs.

The transcript encoding PRPL34 was predicted to be targeted by three ASBVdB-sRNAs, all 21-nt variants of ASBVdB-sRNA39 (a, b, and c), differing only in the two terminal residues at their 3’ ends. These duplexes had exceptionally low E-values of 2.0 under stricter scoring parameters, some of the lowest among our predictions (Supplementary Table S5), indicating greater reliability compared to duplexes formed with *TGD2*, which all had higher E-values of 3.5. This difference was further corroborated by the complementarity patterns in the respective duplexes: in the region of the ASBVdB-sRNA:mRNA target duplexes where the fewest mismatches occur in bona fide miRNA:mRNA hybrids (position 2–13 of the sRNA when reading 5’-3’) [48], *PRPL34* displayed nearly perfect base pairing (with the exception of a single G:U base pair), while *TGD2* had two G:U base pairs in this region and more mismatches overall (Table 2). The occurrence of G:U base pairs in the cleavage site of the sRNA:*TGD2* duplexes (positions 11 and 12 of the respective ASBVdB-sRNAs) indicate reduced likelihood of direct silencing of *TGD2* by ASBVdB-sRNA-induced PTGS. Expression analysis reinforced these findings, showing that *PRPL34* transcript accumulation was reduced by more than twofold in SY samples compared to both SG and AS samples (log_2_FC ≤ -1.2; *p*adj ≤ 0.05). In contrast, suppression of *TGD2* was less pronounced in yellow tissues (log_2_FC ≤ -0.47; *p*adj ≤ 0.1). These data suggest that, while the downregulation of both *PRPL34* and *TGD2* may contribute to bleaching symptoms in ASBVd-infected trees, the evidence more strongly supports the direct targeting of *PRPL34* mRNAs by ASBVdB-sRNAs *in planta*.

## Discussion

In this study, we investigated leaf bleaching in avocado caused by symptomatic ASBVd infection. Our primary goal was to determine whether the molecular mechanism underlying ASBVd pathogenesis aligns with that of other members of the family *Avsunviroidae* or follows a distinct pathway. We confirmed that the pathogenic determinant in the ASBVd genome associated with bleaching symptoms is the addition of at least one uracil residue within positions 115–118 of the viroid RTL, consistent with findings from previous studies [33–35]. Interestingly, unlike PLMVd and CChMVd, where bleaching-associated variants are restricted to yellow tissue [8–10], ASBVd variants containing the pathogenic determinant were detected not only in yellow tissue but also in green sectors of bleached leaves and in asymptomatic (fully green) leaves from symptomatic trees. In contrast, none of the ASBVd variants obtained from asymptomatic leaves of symptomless carrier trees, sequenced in our previous study [37], contained the uracil insertion associated with chlorosis. This suggests that ASBVd genomes containing the pathogenic determinant represent quasispecies of a severe variant necessary for chlorosis to develop, while the presence of only ASBVd-SC variants results in asymptomatic infection. We propose that the onset of sunblotch symptoms in previously asymptomatic ASBVd-infected avocado trees (a situation often observed in avocado orchards with latent ASBVd infections [32, 54, 55]) is driven by mutations within ASBVd-SC genomes, leading to the accumulation of variants containing the pathogenic determinant. Deep sequencing of the quasispecies populations occurring in bleached leaves, as well as investigations into ASBVd sequence variants present in variegated leaves and chlorotic fruit, will further substantiate the association of the severe variant of ASBVd with the occurrence of chlorotic symptoms typical in avocado sunblotch disease.

The presence of ASBVd variant sequences containing the pathogenic determinant in yellow and green tissues of sunblotch-affected trees suggests that pathogenesis of this viroid (genus *Avsunviroid*) differs from that of PLMVd and CChMVd (genus *Pelamoviroid*). Tissue-specific segregation of avsunviroid populations appears to be genus-specific within the family *Avsunviroidae*. Nonetheless, the localised symptoms induced by ASBVd resemble those caused by PLMVd and CChMVd, indicating that the basic molecular mechanism triggering disease symptoms may be similar to that of pelamoviroids [7]. The presence of severe ASBVd sequence variants in both green and yellow tissues of symptomatic avocado trees suggests that RNA silencing of host factors leading to chlorosis occurs due to factors other than the mere presence of ASBVd variants carrying the pathogenic determinant. The observed variations in the viroid titre across the different tissue types in this study hint at the accumulation levels of severe ASBVd variants as a potential determining factor. This finding contrasts with the results of a CChMVd study, which showed no differences in viroid accumulation levels between chlorotic and green tissues of symptomatic chrysanthemum [10].

Using semi-quantitative real-time PCR, we showed that ASBVd accumulation was consistently higher in yellow sectors than in adjacent green sectors of bleached leaves for all four biological replicates, corroborating the findings of sequential PAGE detection in an earlier report [33]. Asymptomatic leaves exhibited the lowest viroid concentrations overall, with two AS samples yielding results indistinguishable from uninfected controls used in the assay. The absence of ASBVd in asymptomatic branches of symptomatic trees has been reported previously [56]. In our analysis, the ASBVd titre was directly correlated with the abundance of vd-sRNAs in the corresponding samples. SY samples exhibited, on average, 90 times more ASBVd-sRNAs than their SG counterparts, normalised to reads per million. In the AS tissues, no ASBVd-sRNAs were found in the two samples in which no viroid was detected by the diagnostic assay (Tree1_AS and Tree 3_AS). For the remaining AS samples, three and 57 vd-sRNAs were found in the Tree 2_AS in Tree 4_AS samples, respectively.

A correlation between viroid titre and ASBVd-sRNA accumulation was also observed in a previous study, in which Northern blot hybridisation revealed significantly higher levels of ASBVd and its associated vd-sRNAs in yellow sectors of bleached leaves compared to green sectors of the same leaves. No ASBVd-sRNAs were detected in symptomless carrier tissues [16]. Our findings strongly support the conclusions in that study, which proposed that increased viroid accumulation provides a higher abundance of templates for processing by DCL RNases, resulting in the generation of more ASBVd-sRNAs [16]. Considering the pathogenesis of other avsunviroids, we hypothesise that the bleaching symptoms caused by ASBVd occur when severe viroid variants accumulate to a critical threshold in leaf tissues, resulting in the production of numerous ASBVd-sRNAs containing the pathogenic determinant, which subsequently target host genes for downregulation by PTGS. An unresolved question is why viroid accumulation levels vary so drastically between different tissues of symptomatic avocado trees, particularly since previous studies have shown that ASBVd is evenly distributed in the branches of symptomless carrier trees [56]. The differences we observed in the secondary structures of the RTL of ASBVd variants associated with symptomatic compared to asymptomatic avocado trees might partially account for this phenomenon, since alterations to the secondary structure of potato spindle tuber viroid (PSTVd) affected the replication and movement of the viroid *in planta* [57]. Future research should include manual inoculation of avocado with natural and artificial variants of ASBVd, where site-directed mutagenesis (such as that performed for PLMVd and CChMVd [8–10]) may reveal the relative importance of alterations to the primary and secondary structure, respectively, of the severe ASBVd variant.

To investigate whether vd-sRNAs arising from the severe ASBVd variant can trigger chlorosis through RNA silencing, we focused on ASBVd-sRNAs derived from the RTL region containing the pathogenic determinant (designated as ASBVdB-sRNAs). Notably, almost no ASBVdB-sRNAs were detected in SG tissues, despite the presence of numerous ASBVd-sRNAs derived from other regions of the viroid. Additionally, although the specific ASBVd-sRNAs present in leaves of symptomless carrier trees remain unknown, the absence of the severe variant (carrying the pathogenic determinant) in those tissues likely corresponds to the absence of associated ASBVdB-sRNAs. For these reasons, we propose that the ASBVdB-sRNAs specifically derived from the severe variant play a pivotal role in inducing chlorosis. Using available transcriptome data, we evaluated the expression of avocado transcripts potentially targeted by ASBVdB-sRNAs and identified 25 significantly downregulated genes (*p*adj ≤ 0.1) in yellow tissues. However, further analysis showed that the majority of these genes were unlikely to be suppressed through viroid-associated PTGS, as there was insufficient evidence supporting the formation of duplexes with ASBVdB-sRNAs. Instead, it is likely that most of the downregulated genes were affected as part of the signalling cascades triggered by the initial molecular alteration following infection by the severe ASBVd variant.

Four candidate genes were identified, with the strongest *in silico*-based evidence of downregulation through PTGS directed by 21- and 22-nt ASBVdB-sRNAs. These genes were further investigated to determine whether their suppression via RNA silencing may be the initial molecular alteration leading to bleaching symptoms. We focused on two genes, *TGD2* and *PRPL34*, present in the nuclear genome of avocado but predicted to encode chloroplastic proteins, as the disrupted function of chloroplastic proteins would contribute directly to chlorosis [8–10]. The function of PRPL34 in arabidopsis has not been determined, but its annotation as a plastid ribosomal protein (PRP) associated with the 50S subunit suggests a role in the translation of chloroplastic transcripts. Investigation of mutants of several other PRPs associated with 50S ribosomes in arabidopsis, rice, and maize demonstrated that reduced expression of these genes caused albinism, yellowing, and/or disruption of photosynthesis [58], likely due to suppressed translation of chloroplastic mRNAs. It is therefore plausible that decreased accumulation of PRPL34 in yellow sectors of ASBVd-infected avocado leaves may interfere with translation in chloroplasts, leading to downstream reduction in the abundance of other chloroplastic proteins. In this way, targeting of *PRPL34* mRNA by ASBVdB-sRNAs for degradation by PTGS could have a cumulative effect, ultimately causing leaf chlorosis characteristic of sunblotch disease.

Although the downregulation of *TGD2* in yellow tissues was less pronounced than that of *PRPL34*, its suppression could explain the ultrastructural changes observed in symptomatic avocado tissues [59]. The arabidopsis TGD2 protein has been identified as a component of a chloroplastic ATP-binding cassette (ABC) transporter essential for transporting lipids from the cytoplasm into chloroplasts, thereby enabling thylakoid biogenesis [53, 60]. In avocado, reduced accumulation of TGD2 protein in chloroplasts would likely result in disruption of thylakoid biogenesis, which would lead to the malformed, diminished or absent grana observed in yellow tissues of symptomatic avocado leaves [59, 61]. This disruption aligns directly with the ultrastructural abnormalities associated with sunblotch symptoms.

In this study, we demonstrate that the suppression of *PRPL34* and *TGD2* correlates with their predicted targeting by ASBVdB-sRNAs. These sRNAs are the correct size (21 and 22 nt) and have the 5’-terminal uracil residue required for loading into AGO1 to facilitate RISC-mediated silencing [26, 51]. The characteristics of these ASBVdB-sRNAs are similar to those of vd-sRNAs implicated in chlorosis during PLMVd and CChMVd infection [8–10]. We observed stronger evidence for ASBVdB-sRNA-guided targeting of *PRPL34* compared to *TGD2*, where duplexes formed with *PRPL34* mRNA had lower E-values (indicating higher reliability) and fewer mismatches overall than those formed with *TGD2* transcripts. It is possible that both predictions are correct and that both genes are downregulated through PTGS by vd-sRNAs but that the weaker duplexes formed with *TGD2* result in less-efficient cleavage of this transcript compared to that of *PRPL34* mRNA. Alternatively, the mismatches located within the cleavage site of the ASBVdB-sRNA:*TGD2* hybrids may suggest that direct targeting of *TGD2* was falsely predicted by our methods and that downregulation of this gene was due to the action of *trans-*acting siRNAs or transcriptional gene silencing, or was a downstream effect of the initial signalling cascades triggered upon infection. This would explain the observed difference in the extent of downregulation between the two genes in yellow tissues, and indicates that downregulation of *PRPL34* due to targeting by ASBVdB-sRNAs is more likely to be the initial molecular lesion triggering bleaching symptoms. Future experiments, such as RNA ligase-mediated random amplification of cDNA ends (RLM-RACE), as performed in PLMVd and CChMVd studies [8–10], will aid in determining which of the predicted ASBVdB-sRNAs guide cleavage of host mRNAs at the expected sites. These experiments will also clarify if decreased *PRPL34* and *TGD2* transcript accumulation is the direct result of RNA silencing occurring in bleached ASBVd-infected tissues.

## Conclusions

This study aimed to broaden the current understanding of ASBVd pathogenesis by analysing the molecular characteristics of bleached leaves in viroid-infected avocado trees. This represents the first examination of ASBVd sequence variants in distinct yellow and green leaf tissues of symptomatic trees. We identified populations of the severe ASBVd variant, characterised by an additional uracil residue in the RTL region of the genome, present across all leaf tissues of symptomatic trees. However, ASBVd accumulation varied significantly, with yellow leaf sectors exhibiting the highest viroid titre and containing more ASBVd-sRNAs compared to green tissues. This is also the first study to sequence sRNAs derived from ASBVd and compare their expression across different tissue types. We postulate that the increased accumulation of severe variant populations in yellow tissues provides more viroid template for DCL cleavage, leading to the production of more vd-sRNAs that induce disease symptoms via RNA silencing. Two chloroplastic mRNAs, *PRPL34* and *TGD2*, were identified as potential targets of ASBVd-sRNAs containing the pathogenic determinant. Their reduced accumulation in yellow tissues correlates with the development of chlorosis in symptomatic avocado leaves, implicating their suppression in the development of sunblotch symptoms. *In silico* analysis provides the strongest evidence for targeting of *PRPL34* by ASBVd-sRNAs during ASBVd infection and suggests that downregulation of *TGD2* may be due to alternative gene silencing mechanisms. Future research is needed to confirm the predicted targeting of *PRPL34* mRNA *in planta* and establish a causal relationship between its downregulation and the yellowing of leaf tissues. Nonetheless, this work provides the first in-depth investigation into the potential role of RNA silencing in ASBVd pathogenesis, illustrating that avocado sunblotch disease is likely driven by vd-sRNA-guided silencing of host factors. Unlike in the case of pelamoviroids, this RNA silencing is not triggered by the presence of specific variants in infected hosts, but is instead associated with the accumulation levels of severe variants in affected tissues. Future research should investigate ASBVd variants in chlorotic and asymptomatic tissues of variegated leaves and symptomatic fruit to further elucidate the role of the severe ASBVd variant in causing chlorosis in sunblotch-affected avocado. These efforts will help clarify the molecular mechanisms underlying chlorosis and the broader impact of ASBVd on avocado health.

## Electronic Supplementary Material

Below is the link to the electronic supplementary material

Supplementary Material 1

## Data Availability

ASBVd variant sequences obtained in this study have been uploaded to the NCBI GenBank database under accession numbers PQ619768-PQ619817. Raw sRNA-sequencing data are available from the Sequence Read Archive of NCBI GenBank under BioProject PRJNA1188101. The mRNA-sequencing data generated and analysed in the current study are not yet publicly available, due to ongoing analysis for publication elsewhere, but are available from the corresponding author on reasonable request.
