# Supplementary material for: Small RNAs derived from avocado sunblotch viroid and their association with bleaching symptoms: implications for pathogenesis in avocado sunblotch disease
Source: Arch Virol. 2025 Sep 11;170(10):205. doi: 10.1007/s00705-025-06360-z (PMC12423203; doi:10.1007/s00705-025-06360-z)
Supplement: Supplementary file 1 — Supplementary Material 1 [file 705_2025_6360_MOESM1_ESM.docx]

**Supplementary Information**

**Small RNAs derived from avocado sunblotch viroid and their association with bleaching symptoms: implications for pathogenesis in avocado sunblotch disease**

**Melissa Joubert^1,2^, Noëlani van den Berg^1,2^, Jacques Theron^1^, Velushka Swart^1,2^***

^1^Department of Biochemistry, Genetics and Microbiology, Faculty of Natural and Agricultural Sciences, University of Pretoria, Pretoria, Gauteng, South Africa

^2^Hans Merensky Chair in Avocado Research, Forestry and Agricultural Biotechnology Institute, University of Pretoria, Pretoria, Gauteng, South Africa

***Correspondence**: [velushka.swart@fabi.up.ac.za](mailto:velushka.swart@fabi.up.ac.za)

**Contents:**

**Fig. S1.** Confirmation of real-time PCR amplification of avocado sunblotch viroid (ASBVd) in 12 samples.

**Fig. S2.** Alignment of avocado sunblotch viroid (ASBVd) clones sequenced in this study with previously characterised variants.

**Fig. S3.** Size distribution of small RNA (sRNA)-sequencing reads in different samples.

**Fig. S4.** Polarity and size distribution of avocado sunblotch viroid-derived small RNAs (ASBVd-sRNAs) in different sample types.

**Fig. S5.** Venn diagram of 44 target genes downregulated in yellow leaf tissues in ASBVd-infected trees displaying bleaching symptoms**.**

**Table S1.** The source of leaf material used in this study.

**Table S2.** Average fluorescence values for real-time PCR performed to detect ASBVd in individual samples.

**Table S3.** Number of small RNA (sRNA) reads derived from avocado sunblotch viroid (ASBVd) in individual samples.

**Table S4.** ASBVd-sRNAs originating from the pathogenic region of bleaching-associated ASBVd variants, sequenced from chlorotic leaf tissues.

**Table S5.** Avocado mRNA targets, predicted to form duplexes with ASBVd-sRNAs, which were downregulated in yellow tissues.


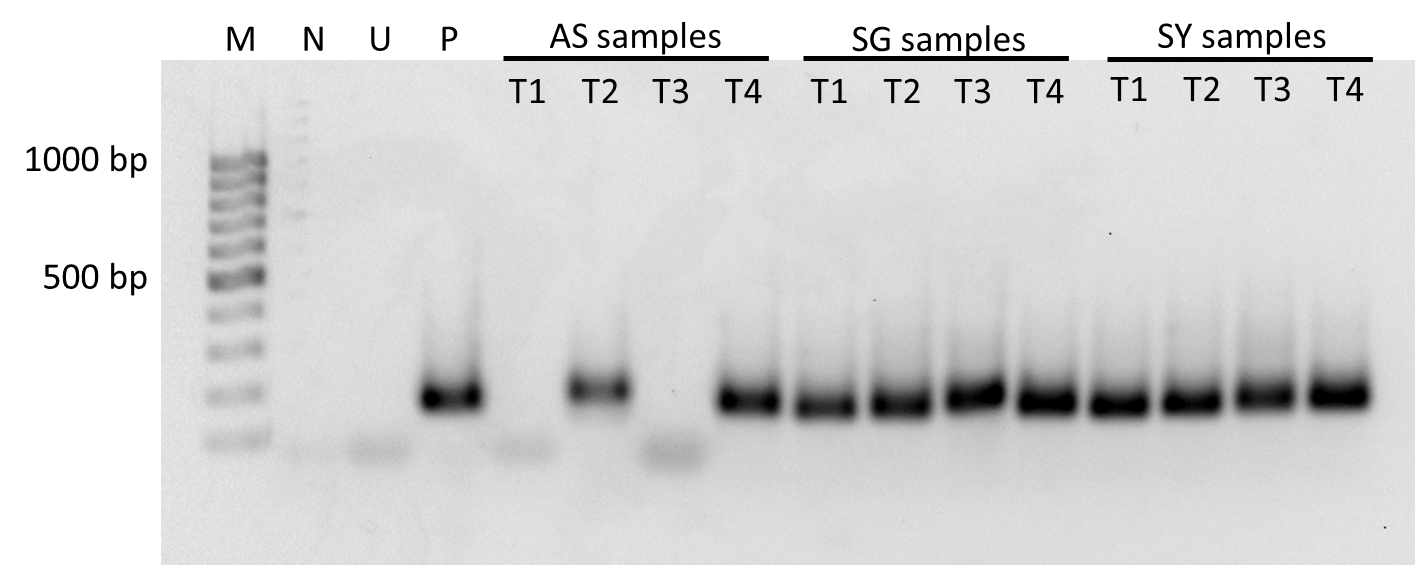


**Fig. S1.** Confirmation of real-time PCR amplification of avocado sunblotch viroid (ASBVd) in 12 samples**.** Products of TaqMan™ amplification of ASBVd in pooled leaf samples harvested from four sunblotch-affected trees (T1-T4), shown on a 2% agarose gel and compared to a subset of controls used in the real-time PCR. The infected control (P) and ten samples that produced fluorescence in the diagnostic assay had single bands with the expected size of ~155 bp. The non-template control (N), uninfected control (U), and two samples that did not fluoresce in the real-time assay had no amplification products. M: 100 bp DNA marker; AS samples: Asymptomatic (fully green) leaves; SG samples: Green sectors of bleached leaves (SG); SY samples: Yellow sectors of bleached leaves.


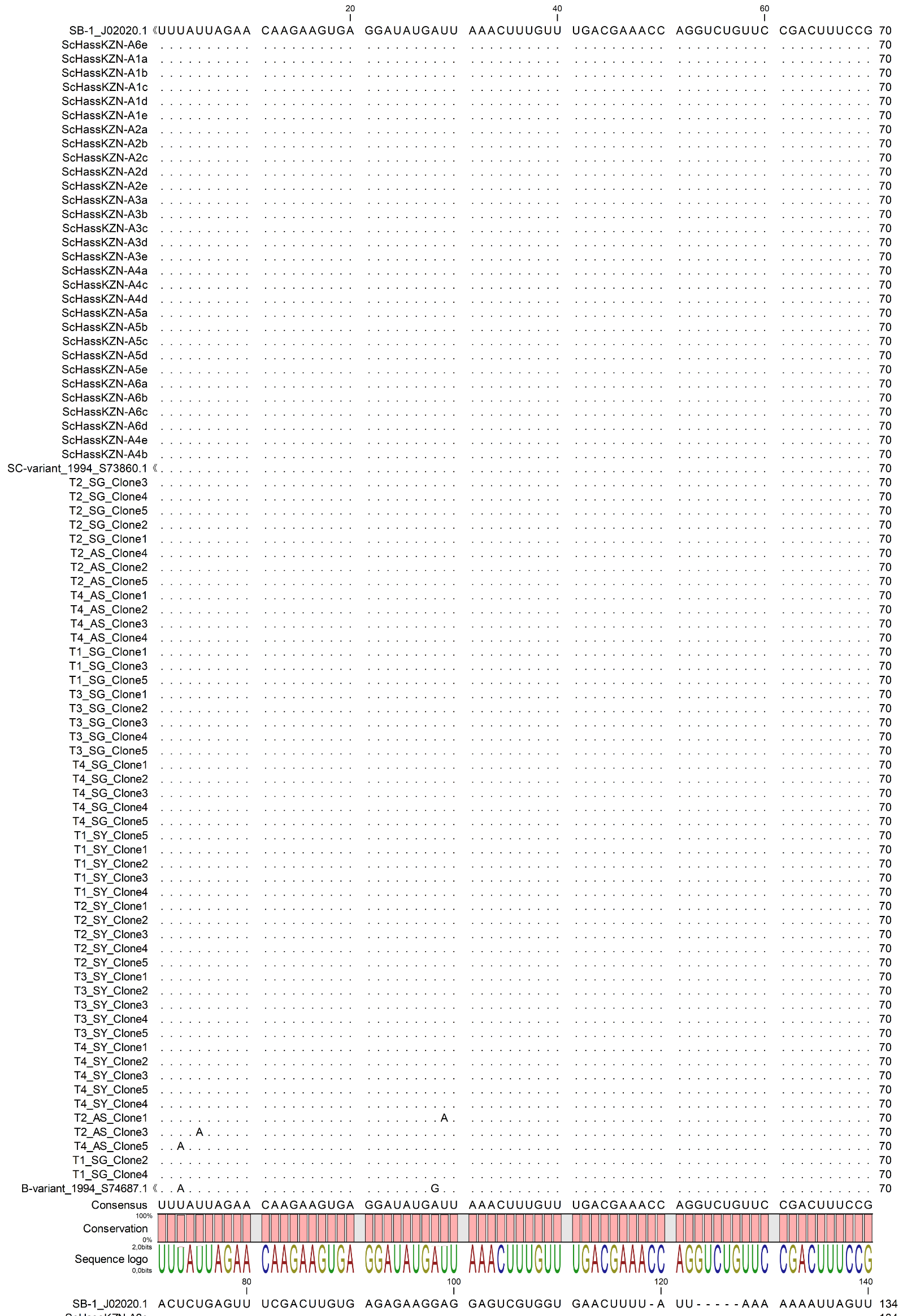


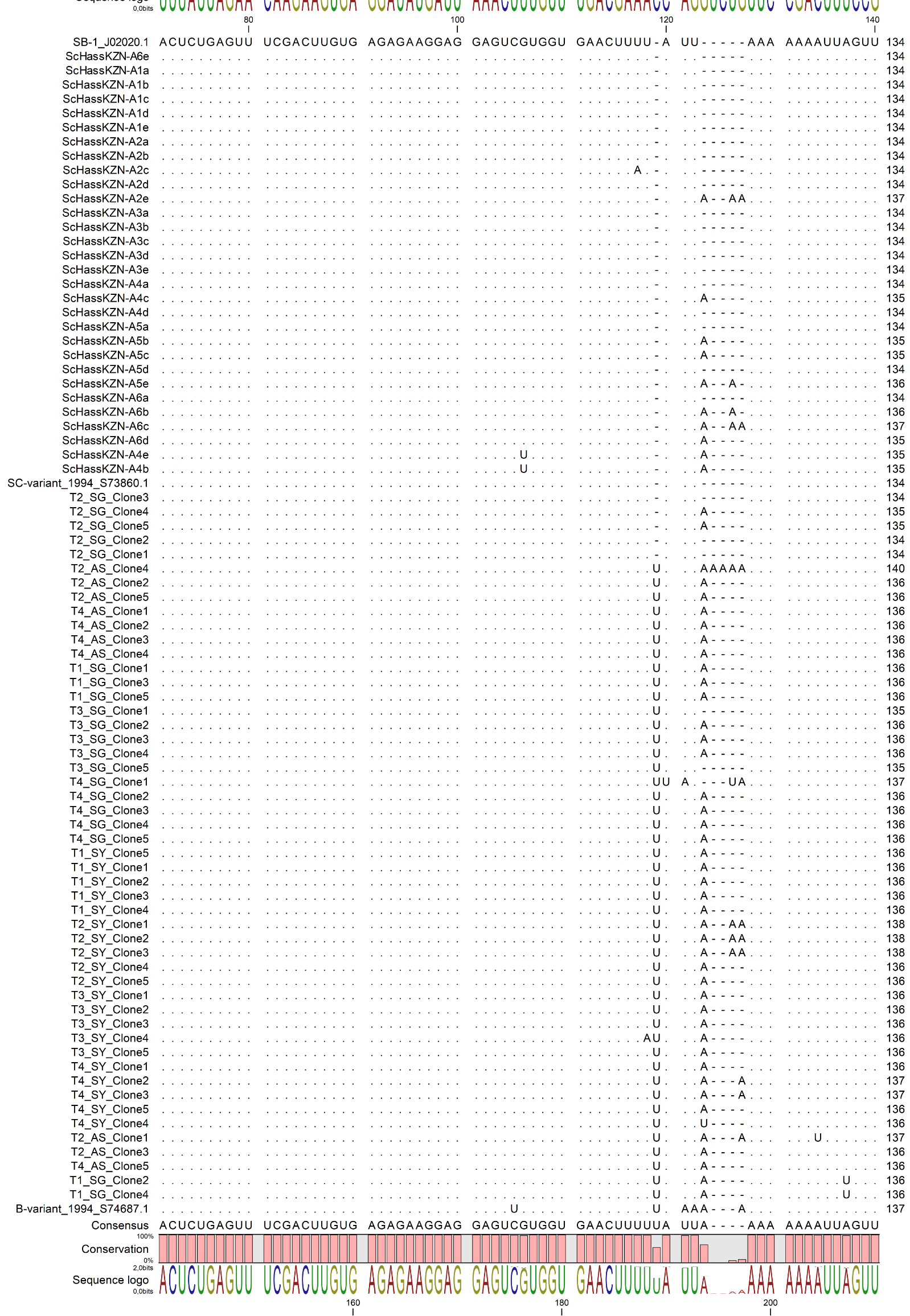


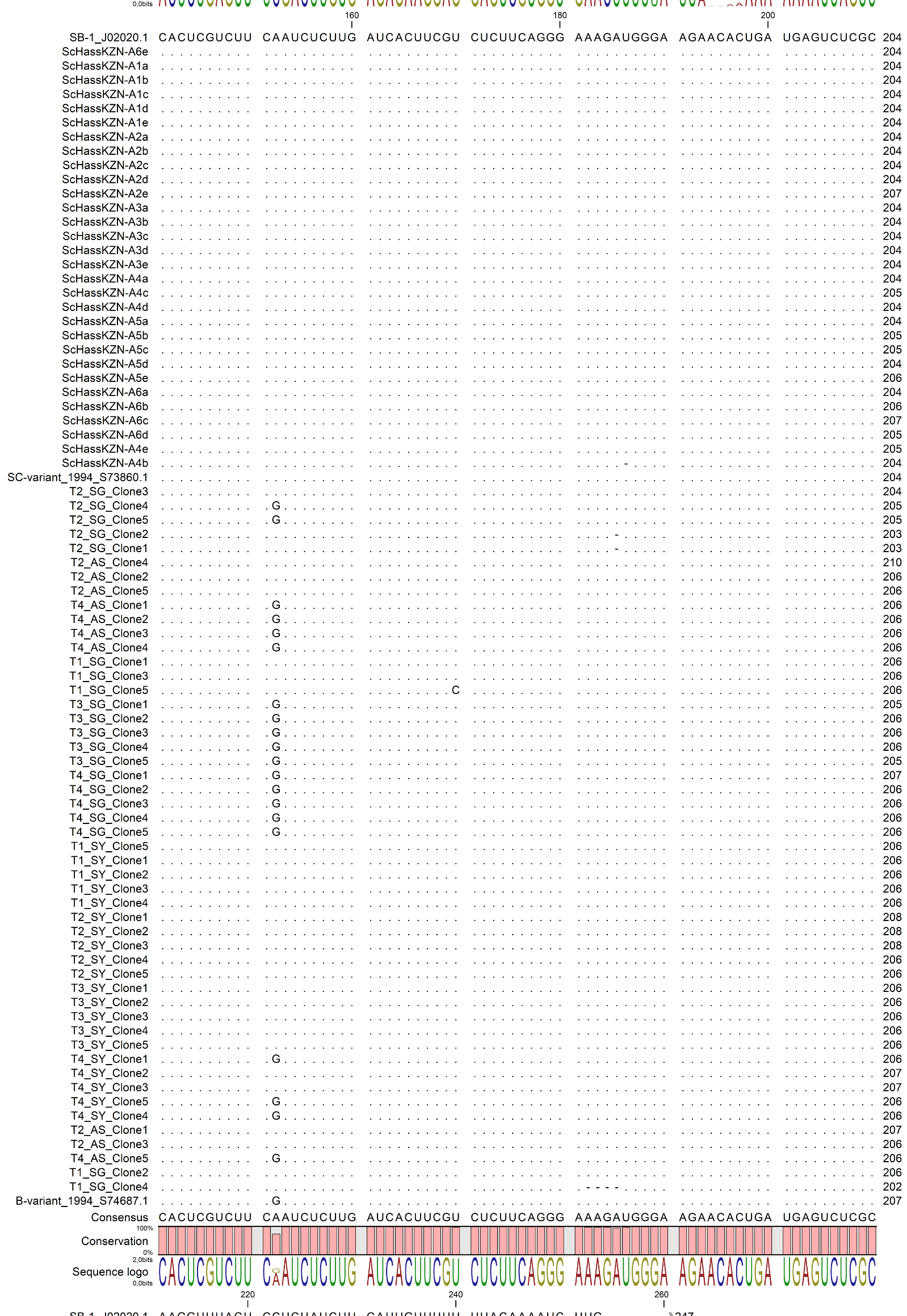


**
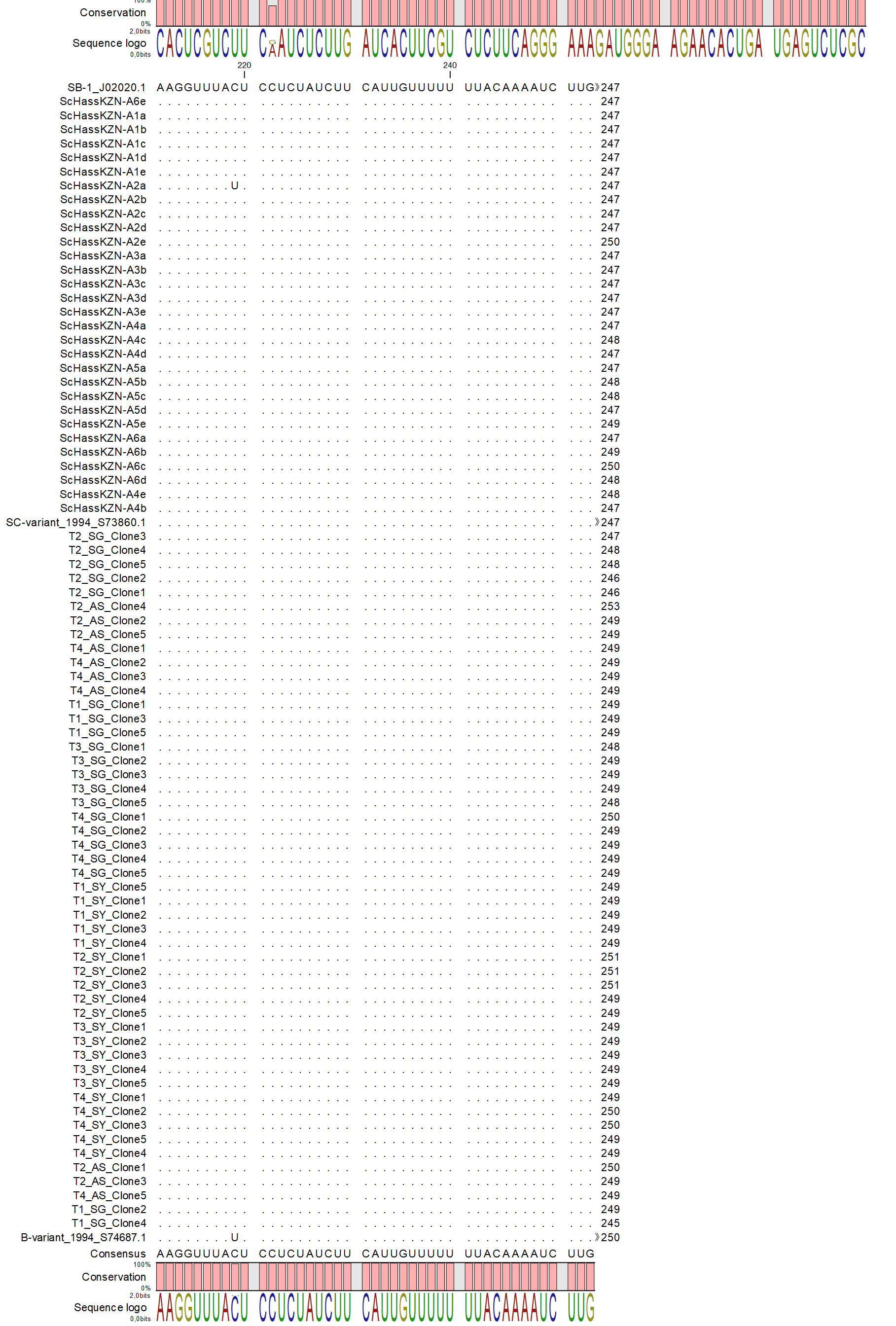
**

**Fig. S2.** Alignment of avocado sunblotch viroid (ASBVd) clones sequenced in this study with previously characterised variants. Variant sequences were obtained from ASBVd isolates cloned from yellow sectors (SY samples) and green sectors (SG samples) of bleached leaves for four biological replicates: Tree 1 (T1), Tree 2 (T2), Tree 3 (T3) and Tree 4 (T4), and from asymptomatic (AS) samples from T2 and T4. The 50 clones sequenced in this study were aligned to the ASBVd type sequence SB-1, the previously characterised bleached (B) and symptomless carrier (SC) variants, and 30 clones sequenced from six asymptomatic ASBVd-infected nursery trees (ScHassKZN A1-A6) in our previous study. The most significant sequence variation was observed at positions 115-128 relative to SB-1 (highlighted by a black border), representing a portion of the right terminal loop (RTL) of the viroid secondary structure. The insertion of at least one uracil residue at positions 115-118 was observed in green and yellow tissues of trees with sunblotch symptoms, but not in asymptomatic (fully green) leaf tissue from symptomless carrier trees.


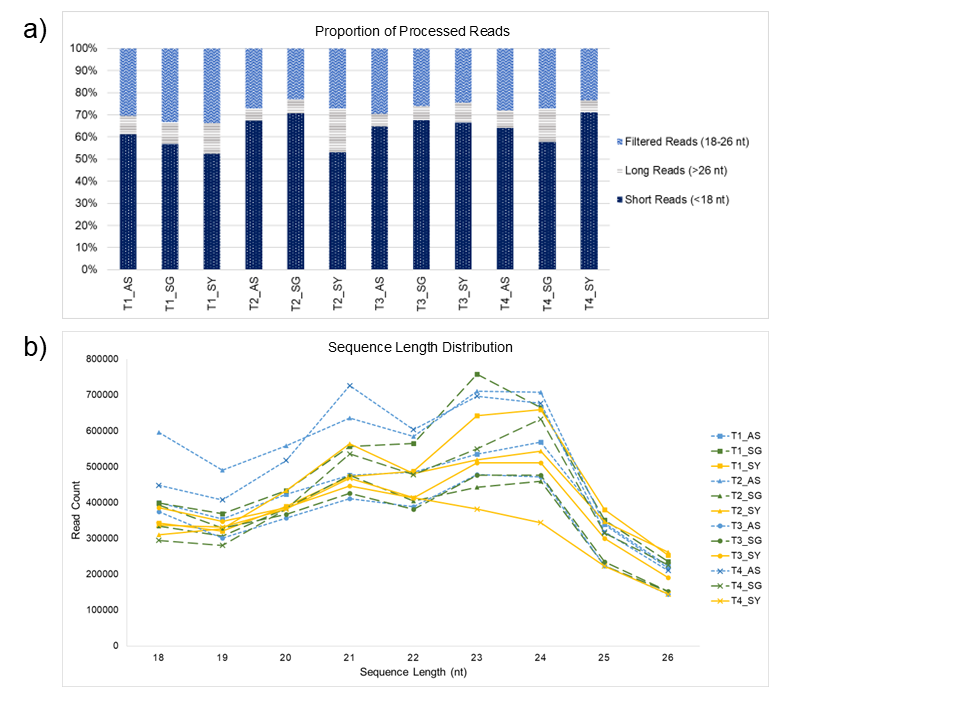


**Fig. S3.** Size distribution of small RNA (sRNA)-sequencing reads in different samples. (**a**) The proportion of reads for each sample that were maintained for further analysis (average 27.8%), reads discarded for being too short (average 62.7%) and discarded reads that were too long (average 9.5%). (**b**) The sequence length distribution for filtered reads maintained for further analysis (18-26 nt). Data is shown for three sample types (asymptomatic (AS), green sectors of bleached leaves (SG), and yellow sectors of bleached leaves (SY)) for four biological replicates (Tree 1-4 (T1-T4)).


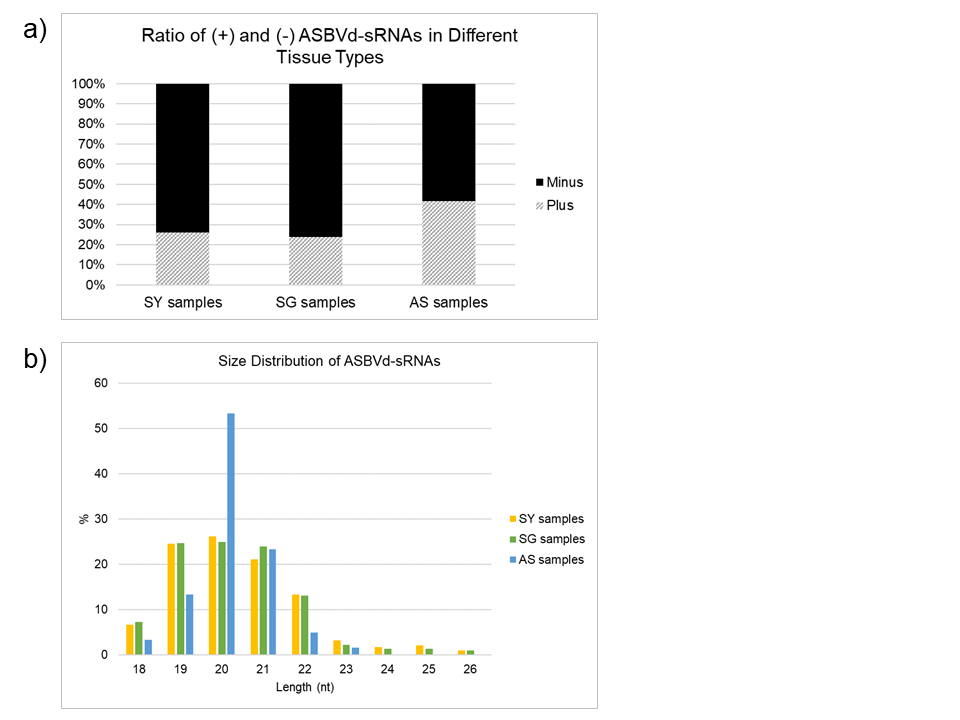


**Fig. S4.** Polarity and size distribution of avocado sunblotch viroid-derived small RNAs (ASBVd-sRNAs) in different sample types. (**a**) The proportion of ASBVd-sRNAs derived from the sense (plus; +) and antisense (minus; -) strands of the ASBVd genome according to tissue type. (**b**) The percentage of ASBVd-sRNAs corresponding to specific sizes within assorted sample types. AS samples were obtained from asymptomatic (fully green) leaves from symptomatic trees, while SG and SY samples consist of material from the green and yellow sectors of bleached leaves, respectively.


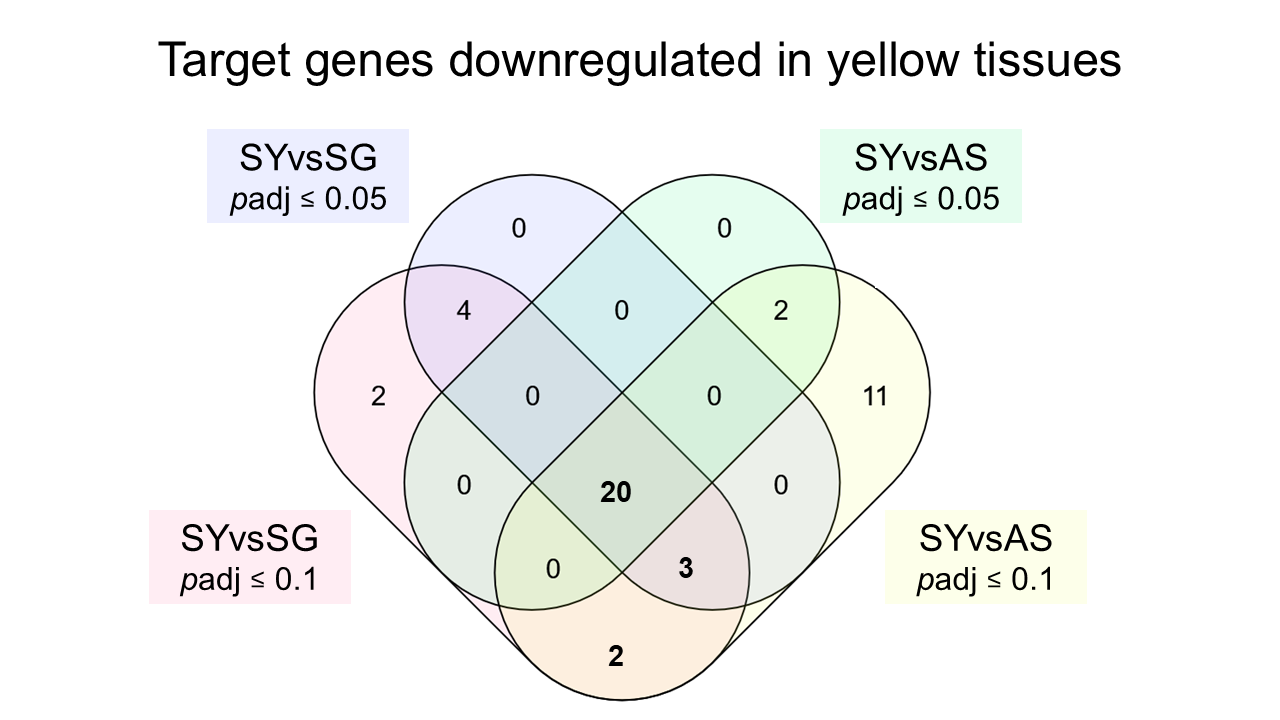


**Fig. S5.** Venn diagram of 44 target genes downregulated in yellow leaf tissues in ASBVd-infected trees displaying bleaching symptoms**.** The genes were predicted as targets of avocado sunblotch viroid-derived small RNAs (ASBVd-sRNAs) for post-transcriptional gene silencing by *in silico* analysis. Putative targets were all significantly downregulated (*p*adj ≤ 0.1) in yellow sectors of bleached leaves (SY) relative to either green sectors of bleached leaves (SG) or asymptomatic leaves from symptomatic trees (AS). Genes downregulated in both SY vs. SG and SY vs. AS are indicated in bold. No targets were significantly downregulated in SG vs. AS.

**Table S1.** The source of leaf material used in this study.

| Biological replicate | Cultivar^a^ | Bleached leaves present | Variegated leaves present | Chlorotic fruit present^b^ | Number of bleached leaves pooled for SY and SG samples^c^ | Number of asymptomatic (fully green) leaves pooled for AS samples^c^ |
| --- | --- | --- | --- | --- | --- | --- |
| Tree 1 | A | Yes | Yes | No | 30 | 5 |
| Tree 2 | B | Yes | Yes | Yes | 22 | 5 |
| Tree 3 | B | Yes | Yes | No | 14 | 5 |
| Tree 4 | C | Yes | Yes | Yes | 19 | 5 |

^a^Samples were collected under a proprietary agreement with South African avocado growers. Individual cultivars are therefore indicated by different letters.

^b^All trees sampled were in fruit at the time of collection, though some trees did not have visible fruit symptoms.

^c^All leaves collected were fully developed at the time of sampling.

AS: material obtained from asymptomatic (fully green) leaves; SG: material obtained from the green sectors of bleached leaves; SY: material obtained from the yellow sectors of bleached leaves

**Table S2.** Average fluorescence values for real-time PCR performed to detect ASBVd in individual samples.

| Sample Name | Sample Contents | Average Ct Value  (3 technical replicates) |
| --- | --- | --- |
| NTC | Non-template control | N/A |
| cDNA NTC - Y | Non-template control for viroid cDNA synthesis from yellow samples | N/A |
| PreAmp NTC - Y | Non-template control for pre-amplification reaction for yellow samples | N/A |
| cDNA NTC PreAmp - Y | Non-template control for viroid cDNA synthesis,  pre-amplified with yellow samples | N/A |
| Neg - Y | Known uninfected – cDNA synthesised and pre-amplified  with yellow samples | N/A |
| Pos - Y | Known infected (symptomatic – bleached leaves) – cDNA synthesised and pre-amplified with yellow samples | 29.40 |
| cDNA NTC - G | Non-template control for viroid cDNA synthesis from green samples | N/A |
| PreAmp NTC - G | Non-template control for pre-amplification reaction for green samples | N/A |
| cDNA NTC PreAmp - G | Non-template control for viroid cDNA synthesis,  pre-amplified with green samples | N/A |
| Neg - G | Known uninfected – cDNA synthesised and pre-amplified  with green samples | N/A |
| Pos - G | Known infected (asymptomatic) – cDNA synthesised and pre-amplified with green samples | 29.71 |
| Tree1_AS | Asymptomatic leaves – biological replicate 1 | N/A |
| Tree2_AS | Asymptomatic leaves – biological replicate 2 | 37.12 |
| Tree3_AS | Asymptomatic leaves – biological replicate 3 | N/A |
| Tree4_AS | Asymptomatic leaves – biological replicate 4 | 30.15 |
| Tree1_SG | Green sectors of bleached leaves – biological replicate 1 | 28.00 |
| Tree2_SG | Green sectors of bleached leaves – biological replicate 2 | 26.18 |
| Tree3_SG | Green sectors of bleached leaves – biological replicate 3 | 30.37 |
| Tree4_SG | Green sectors of bleached leaves – biological replicate 4 | 26.62 |
| Tree1_SY | Yellow sectors of bleached leaves – biological replicate 1 | 17.39 |
| Tree2_SY | Yellow sectors of bleached leaves – biological replicate 2 | 17.42 |
| Tree3_SY | Yellow sectors of bleached leaves – biological replicate 3 | 19.02 |
| Tree4_SY | Yellow sectors of bleached leaves – biological replicate 4 | 19.86 |

N/A – No fluorescence detected after 40 amplification cycles

**Table S3.** Number of small RNA (sRNA) reads derived from avocado sunblotch viroid (ASBVd) in individual samples.

| Sample Type | Biological Replicate | Number of raw sRNA reads | Number of reads after filtering | Number of ASBVd-sRNAs in filtered library | ASBVd-sRNAs per million filtered reads |
| --- | --- | --- | --- | --- | --- |
| **AS** | Tree1 | 12,490,597 | 3,806,703 | 0 | 0.00 |
|  | Tree2 | 17,810,540 | 4,840,219 | 3 | 0.62 |
|  | Tree3 | 10,628,969 | 3,149,350 | 0 | 0.00 |
|  | Tree4 | 16,507,218 | 4,606,403 | 57 | 12.37 |
| **SG** | Tree1 | 13,005,385 | 4,334,959 | 227 | 52.36 |
|  | Tree2 | 13,890,995 | 3,184,772 | 684 | 214.77 |
|  | Tree3 | 12,420,530 | 3,236,852 | 79 | 24.41 |
|  | Tree4 | 13,565,594 | 3,696,011 | 334 | 90.37 |
| **SY** | Tree1 | 11,682,135 | 3,949,789 | 29,914 | 7,573.57 |
|  | Tree2 | 13,970,063 | 3,787,878 | 38,000 | 10,032.00 |
|  | Tree3 | 14,235,126 | 3,491,534 | 29,203 | 8,363.95 |
|  | Tree4 | 12,958,727 | 3,026,825 | 25,725 | 8,499.00 |

AS: Asymptomatic (fully green) leaves from symptomatic trees; SG: Green sectors of bleached leaves from symptomatic trees; SY: Yellow sectors of bleached leaves from symptomatic trees

**Table S4.** ASBVd-sRNAs originating from the pathogenic region of bleaching-associated ASBVd variants, sequenced from yellow leaf tissues.

| vd-sRNA name | Length (nt) | ASBVdB-sRNA sequence^a^ | sRNA reads^b^ | Viroid strand^c^ | 5' position^d^ |
| --- | --- | --- | --- | --- | --- |
| ASBVdB-sRNA1 | 25 | AAGGAGGAGUCGUGGUGAACUUUUU | 1 | (+) | 95 |
| ASBVdB-sRNA2 | 26 | UAAGGAGGAGUCGUGGUGAACUUUUU | 1 | (+) | 94 |
| ASBVdB-sRNA3 | 24 | AGGAGGAGUCGUGGUGAACUUUUU | 1 | (+) | 96 |
| ASBVdB-sRNA4 | 26 | AGGAGGAGUCGUGGUGAACUUUUUAU | 2 | (+) | 96 |
| ASBVdB-sRNA5a | 26 | GGAGGAGUCGUGGUGAACUUUUUAUU | 9 | (+) | 97 |
| ASBVdB-sRNA5b | 26 | GGAGGAGUCGUGGUGAACUUUUUACU | 1 | (+) | 97 |
| ASBVdB-sRNA5c | 26 | GGAGGAGUCGUGGUGAACUUUUUAUG | 2 | (+) | 97 |
| ASBVdB-sRNA5d | 26 | GGAGGAGUCGUGGUGAACUUUUUAUC | 5 | (+) | 97 |
| ASBVdB-sRNA5e | 26 | GGAGGAGUCGUGGUGAACUUUAUAUC | 1 | (+) | 97 |
| ASBVdB-sRNA5f | 26 | GGAGGAGUCGUGGUGAACUUUAUAAU | 1 | (+) | 97 |
| ASBVdB-sRNA6a | 25 | GGAGGAGUCGUGGUGAACUUUUUAU | 2 | (+) | 97 |
| ASBVdB-sRNA6b | 25 | GGAGGAGUCGUGGUGAACUUUAUAC | 1 | (+) | 97 |
| ASBVdB-sRNA7a | 25 | GAGGAGUCGUGGUGAACUUUUUAUU | 1 | (+) | 98 |
| ASBVdB-sRNA7b | 25 | GAGGAGUCGUGGUGAACUUUUUAUC | 1 | (+) | 98 |
| ASBVdB-sRNA8 | 24 | GAGGAGUCGUGGUGAACUUUUUAC | 1 | (+) | 98 |
| ASBVdB-sRNA9a* | 24 | AGGAGUCGUGGUGAACUUUUUAUU | 1 | (+) | 99 |
| ASBVdB-sRNA9b* | 24 | AGGAGUCGUGGUGAACUUUAUAUC | 1 | (+) | 99 |
| ASBVdB-sRNA10* | 23 | AGGAGUCGUGGUGAACUUUUUAC | 1 | (+) | 99 |
| ASBVdB-sRNA11a* | 23 | GGAGUCGUGGUGAACUUUUUAUC | 2 | (+) | 100 |
| ASBVdB-sRNA11b* | 23 | GGAGUCGUGGUGAACUUUUUAUU | 6 | (+) | 100 |
| ASBVdB-sRNA11c* | 23 | CGAGUCGUGGUGAACUUUUUAUU | 1 | (+) | 100 |
| ASBVdB-sRNA12* | 20 | GGAGUCGUGGUGAACUUUUU | 1 | (+) | 100 |
| ASBVdB-sRNA13* | 22 | GGAGUCGUGGUGAACUUUUUAU | 3 | (+) | 100 |
| ASBVdB-sRNA14a* | 22 | GAGUCGUGGUGAACUUUUUACU | 1 | (+) | 101 |
| ASBVdB-sRNA14b* | 22 | GAGUCGUGGUGAACUUUUUAUU | 6 | (+) | 101 |
| ASBVdB-sRNA15 | 23 | AACUUUUUAUUAAAAAAAAUUGG | 1 | (+) | 112 |
| ASBVdB-sRNA16a* | 21 | AGUCGUGGUGAACUUUUUAUC | 1 | (+) | 102 |
| ASBVdB-sRNA16b* | 21 | AGUCGUGGUGAACUUUUUAUU | 2 | (+) | 102 |
| ASBVdB-sRNA16c | 21 | AGUCGUGGUGAACUUUAUAUU | 3 | (+) | 102 |
| ASBVdB-sRNA17* | 25 | AGUCGUGGUGAACUUUUUAUUAAAU | 1 | (+) | 102 |
| ASBVdB-sRNA18* | 20 | AGUCGUGGUGAACUUUUUAU | 1 | (+) | 102 |
| ASBVdB-sRNA19a | 20 | GUCGUGGUGAACUUUUUAUC | 1 | (+) | 103 |
| ASBVdB-sRNA19b | 20 | GUCGUGGUGAACUUUUUAUU | 4 | (+) | 103 |
| ASBVdB-sRNA20a | 19 | UCGUGGUGAACUUUUUAUU | 2 | (+) | 104 |
| ASBVdB-sRNA20b* | 19 | UCGUGGUGAACUUUAUAUU | 2 | (+) | 104 |
| ASBVdB-sRNA21a | 18 | CGUGGUGAACUUUUUAUU | 6 | (+) | 105 |
| ASBVdB-sRNA21b | 18 | CGUGGUGAACUUUUUAUC | 1 | (+) | 105 |
| ASBVdB-sRNA21c | 18 | CGUGGUGAACUUUUUAUG | 1 | (+) | 105 |
| ASBVdB-sRNA21d | 18 | CGUGGUGAACUUUAUAUU | 1 | (+) | 105 |
| ASBVdB-sRNA22 | 20 | CGUGGUGAACUUUUUAUUAG | 1 | (+) | 105 |
| ASBVdB-sRNA23* | 20 | UUUAAUAAAAAGUUCACCAC | 4 | (-) | 125 |
| ASBVdB-sRNA24* | 25 | UAAUAAAGUUCACCACGACUCCUCC | 1 | (-) | 121 |
| ASBVdB-sRNA25 | 24 | UAAAAAGUUCACCACGACUCCUCC | 1 | (-) | 120 |
| ASBVdB-sRNA26a | 25 | AUAAAAAGUUCACCACGACUCCUCC | 1 | (-) | 121 |
| ASBVdB-sRNA26b | 25 | AUAUAAAGUUCACCACGACUCCUCC | 1 | (-) | 121 |
| ASBVdB-sRNA27 | 24 | AUAAAAAGUUCACCACGACUCCUC | 1 | (-) | 121 |
| ASBVdB-sRNA28 | 22 | UUAUAAAGUUCACCACGACUCC | 1 | (-) | 121 |
| ASBVdB-sRNA29 | 23 | AUAUAAAGUUCACCACGACUCCC | 1 | (-) | 121 |
| ASBVdB-sRNA30 | 21 | AUAAAAAGUUCACCACGACUC | 1 | (-) | 121 |
| ASBVdB-sRNA31 | 20 | AAAAAGUUCACCACGACUUC | 3 | (-) | 119 |
| ASBVdB-sRNA32* | 22 | UAAUAAAAAGUUCACCACGACU | 1 | (-) | 123 |
| ASBVdB-sRNA33 | 20 | AUAAAAAGUUCACCACGACC | 2 | (-) | 121 |
| ASBVdB-sRNA34* | 21 | UAAUAAAAAGUUCACCACGAC | 5 | (-) | 123 |
| ASBVdB-sRNA35* | 22 | UUAAUAAAAAGUUCACCACGAC | 1 | (-) | 124 |
| ASBVdB-sRNA36 | 18 | AAUAAAAAGUUCACCACG | 1 | (-) | 122 |
| ASBVdB-sRNA37* | 19 | UAAUAAAAAGUUCACCACG | 1 | (-) | 123 |
| ASBVdB-sRNA38a* | 20 | UUAAUAAAAAGUUCACCACG | 6 | (-) | 124 |
| ASBVdB-sRNA38b | 20 | UUAAUAUAAAGUUCACCACG | 1 | (-) | 124 |
| ASBVdB-sRNA39a* | 21 | UUUAAUAAAAAGUUCACCACG | 5 | (-) | 125 |
| ASBVdB-sRNA39b* | 21 | UUUAAUAAAAAGUUCACCACC | 1 | (-) | 125 |
| ASBVdB-sRNA39c* | 21 | UUUAAUAAAAAGUUCACCAUG | 1 | (-) | 125 |
| ASBVdB-sRNA40 | 18 | UAAUAAAAAGUUCACCAC | 3 | (-) | 123 |
| ASBVdB-sRNA41a* | 19 | UUAAUAAAAAGUUCACCAC | 3 | (-) | 124 |
| ASBVdB-sRNA41b* | 19 | UAAAUAAAAAGUUCACCAC | 1 | (-) | 124 |

^a^The sequences of ASBVd-sRNAs originating from the right terminal loop (RTL) of the viroid genome, where the pathogenic region is underlined for ASBVdB-sRNAs containing the pathogenic determinant of bleaching symptoms.

^b^Number of reads found in yellow sectors of bleached leaves (SY samples).

^c^The strand of the viroid genome from which each sRNA originated; where (+) indicates sRNAs derived from the sense strand of the genome, and (-) sRNAs are generated from the antisense ASBVd genome.

^d^The position on the ASBVd genome to which the 5’ terminus of each sRNA is mapped.

*ASBVdB-sRNAs predicted to target one or more of the seven avocado mRNA candidates with the strongest evidence for duplex formation.

**Table S5.** Avocado mRNA targets, predicted to form duplexes with ASBVdB-sRNAs, which were downregulated in yellow tissues.

| Target gene ID^a^ | Target annotation^a^ | Log2FC - SYvsSG^b^ | *p*adj - SYvSG^b^ | Log2FC - SYvsAS^c^ | *p*adj - SYvsAS^c^ | Target localisation^d^ | vd-sRNA name | vd-sRNA length (nt) | Duplex score - Default^e^ | Duplex score - Strict^f^ | Minimum free energy (kcal/mol)^g^ | Pathogenic determinant within duplex?^h^ |
| --- | --- | --- | --- | --- | --- | --- | --- | --- | --- | --- | --- | --- |
| C01g021570 | Cytochrome P450 94A1 | -1.33 | 0.05 | -1.40 | 0.05 | Endoplasmic reticulum | ASBVdB-sRNA14b | 22 | 2.5 | 2.5 | -28.9 | Yes |
|  |  |  |  |  |  |  | ASBVdB-sRNA11b | 23 | 2.5 | 2.5 | -30.5 | Yes |
|  |  |  |  |  |  |  | ASBVdB-sRNA13 | 22 | 2.5 | 2.5 | -30.5 | Yes |
|  |  |  |  |  |  |  | ASBVdB-sRNA14a | 22 | 2.5 | 2.5 | -29.1 | Yes |
|  |  |  |  |  |  |  | ASBVdB-sRNA11a | 23 | 2.5 | 2.5 | -30.5 | Yes |
|  |  |  |  |  |  |  | ASBVdB-sRNA12 | 20 | 2.5 | 2.5 | -29.8 | Yes |
|  |  |  |  |  |  |  | ASBVdB-sRNA11c | 23 | 3.0 | 3.0 | -29.2 | Yes |
|  |  |  |  |  |  |  | ASBVdB-sRNA16b | 21 | 3.5 | 3.5 | -25.4 | Yes |
|  |  |  |  |  |  |  | ASBVdB-sRNA16a | 21 | 3.5 | 3.5 | -25.4 | Yes |
|  |  |  |  |  |  |  | ASBVdB-sRNA17 | 25 | 3.5 | 3.5 | -25.4 | Yes |
|  |  |  |  |  |  |  | ASBVdB-sRNA18 | 20 | 3.5 | 3.5 | -25.4 | Yes |
|  |  |  |  |  |  |  | ASBVdB-sRNA9a | 24 | 3.5 | 3.5 | -30.8 | Yes |
|  |  |  |  |  |  |  | ASBVdB-sRNA9b | 24 | 3.5 | 3.5 | -28.5 | Yes |
|  |  |  |  |  |  |  | ASBVdB-sRNA10 | 23 | 3.5 | 3.5 | -30.8 | Yes |
| C11g017560 | Glycosyltransferase | -1.00 | 2.748E-04 | -1.32 | 2.857E-08 | Cytoplasm | ASBVdB-sRNA38a | 20 | 2.0 | 2.0 | -18.3 | Yes |
|  |  |  |  |  |  |  | ASBVdB-sRNA35 | 22 | 2.0 | 2.0 | -20.1 | Yes |
|  |  |  |  |  |  |  | ASBVdB-sRNA41a | 19 | 2.0 | 2.0 | -16.6 | Yes |
|  |  |  |  |  |  |  | ASBVdB-sRNA23 | 20 | 2.5 | 2.5 | -17.2 | Yes |
|  |  |  |  |  |  |  | ASBVdB-sRNA39a | 21 | 2.5 | 2.5 | -18.9 | Yes |
|  |  |  |  |  |  |  | ASBVdB-sRNA39b | 21 | 2.5 | 2.5 | -18.0 | Yes |
|  |  |  |  |  |  |  | ASBVdB-sRNA39c | 21 | 2.5 | 2.5 | -17.3 | Yes |
|  |  |  |  |  |  |  | ASBVdB-sRNA34 | 21 | 3.0 | 3.5 | 0.0 | Yes |
|  |  |  |  |  |  |  | ASBVdB-sRNA32 | 22 | 3.0 | 3.5 | 0.0 | Yes |
|  |  |  |  |  |  |  | ASBVdB-sRNA37 | 19 | 3.0 | 3.5 | 0.0 | Yes |
|  |  |  |  |  |  |  | ASBVdB-sRNA38b | 20 | 3.5 | 4.0 | 0.0 | Yes |
|  |  |  |  |  |  |  | ASBVdB-sRNA41b | 19 | 3.5 | 4.0 | 0.0 | Yes |
| C03g004440 | Ammonium_transp domain-containing protein | -2.19 | 0.01 | -1.50 | 0.13 | Cytoplasm | ASBVdB-sRNA12 | 20 | 3.5 | 4.0 | 0.0 | Yes |
| C12g016840 | protein TRIGALACTOSYLDIACYLGLYCEROL 2, chloroplastic | -0.57 | 0.03 | -0.47 | 0.06 | Plastid | ASBVdB-sRNA38a | 20 | 3.5 | 3.5 | -22.3 | Yes |
|  |  |  |  |  |  |  | ASBVdB-sRNA35 | 22 | 3.5 | 3.5 | -23.8 | Yes |
|  |  |  |  |  |  |  | ASBVdB-sRNA41a | 19 | 3.5 | 3.5 | -20.6 | Yes |
|  |  |  |  |  |  |  | ASBVdB-sRNA34 | 21 | 3.5 | 3.5 | -23.6 | Yes |
|  |  |  |  |  |  |  | ASBVdB-sRNA32 | 22 | 3.5 | 3.5 | -26.1 | Yes |
|  |  |  |  |  |  |  | ASBVdB-sRNA37 | 19 | 3.5 | 3.5 | -22.1 | Yes |
|  |  |  |  |  |  |  | ASBVdB-sRNA26a | 25 | 3.5 | 4.0 | 0.0 | Yes |
|  |  |  |  |  |  |  | ASBVdB-sRNA27 | 24 | 3.5 | 4.0 | 0.0 | Yes |
|  |  |  |  |  |  |  | ASBVdB-sRNA30 | 21 | 3.5 | 4.0 | 0.0 | Yes |
|  |  |  |  |  |  |  | ASBVdB-sRNA33 | 20 | 3.5 | 4.0 | No duplex predicted | Yes |
| C07g020980 | DNA polymerase I A, chloroplastic | -0.60 | 0.04 | -0.55 | 0.14 | Plastid | ASBVdB-sRNA23 | 20 | 3.5 | 4.0 | 0.0 | Yes - with mismatch |
|  |  |  |  |  |  |  | ASBVdB-sRNA39a | 21 | 3.5 | 4.0 | 0.0 | Yes - with mismatch |
|  |  |  |  |  |  |  | ASBVdB-sRNA39b | 21 | 3.5 | 4.0 | 0.0 | Yes - with mismatch |
|  |  |  |  |  |  |  | ASBVdB-sRNA39c | 21 | 3.5 | 4.0 | 0.0 | Yes - with mismatch |
| C08g027830 | O-fucosyltransferase family protein | -0.40 | 0.04 | -0.49 | 2.435E-03 | Golgi apparatus | ASBVdB-sRNA23 | 20 | 3.5 | 4.0 | 0.0 | Yes - with mismatch |
|  |  |  |  |  |  |  | ASBVdB-sRNA39a | 21 | 3.5 | 4.0 | 0.0 | Yes - with mismatch |
|  |  |  |  |  |  |  | ASBVdB-sRNA39b | 21 | 3.5 | 4.0 | 0.0 | Yes - with mismatch |
|  |  |  |  |  |  |  | ASBVdB-sRNA39c | 21 | 3.5 | 4.0 | 0.0 | Yes - with mismatch |
| C01g008790 | ABC transporter-like | -0.90 | 1.908E-05 | -1.13 | 2.411E-11 | Plastid | ASBVdB-sRNA34 | 21 | 3.5 | 4.0 | 0.0 | Yes |
|  |  |  |  |  |  |  | ASBVdB-sRNA32 | 22 | 3.5 | 4.0 | 0.0 | Yes |
|  |  |  |  |  |  |  | ASBVdB-sRNA37 | 19 | 3.5 | 4.0 | 0.0 | Yes |
|  |  |  |  |  |  |  | ASBVdB-sRNA26a | 25 | 3.5 | 4.0 | 0.0 | Yes |
|  |  |  |  |  |  |  | ASBVdB-sRNA27 | 24 | 3.5 | 4.0 | 0.0 | Yes |
|  |  |  |  |  |  |  | ASBVdB-sRNA30 | 21 | 3.5 | 4.0 | 0.0 | Yes |
|  |  |  |  |  |  |  | ASBVdB-sRNA33 | 20 | 3.5 | 4.0 | 0.0 | Yes |
| C06g011910 | Ribokinase | -0.53 | 0.03 | -0.61 | 7.680E-04 | Plastid | ASBVdB-sRNA22 | 20 | 3.5 | 4.0 | -16.7 | Partly - not relevant |
| C00g040390 | Unknown protein | -2.06 | 0.01 | -3.46 | 1.306E-03 | Cytoplasm /Nucleus | ASBVdB-sRNA1 | 25 | 3.5 | 3.5 | -27.3 | No |
| C01g034200 | Protein SPIRRIG | -1.76 | 0.05 | -1.98 | 0.02 | Lysosome /Vacuole | ASBVdB-sRNA1 | 25 | 3.5 | 3.5 | -32.7 | Partly - not relevant |
| C08g004490 | Guard cell S-type anion channel SLAC1 | -1.95 | 1.189E-05 | -2.14 | 5.269E-08 | Cell membrane /Lysosome /Vacuole | ASBVdB-sRNA24 | 25 | 3.5 | 4.0 | 0.0 | Yes - with mismatch |
| C05g020570 | Thioredoxin domain-containing protein | -1.02 | 3.477E-03 | -1.16 | 1.471E-04 | Plastid | ASBVdB-sRNA20b | 19 | 3.5 | 3.5 | -29.2 | Yes |
| C09g002300 | 50S ribosomal protein L34, chloroplastic | -1.20 | 2.022E-03 | -1.36 | 5.758E-05 | Plastid | ASBVdB-sRNA23 | 20 | 2.0 | 2.0 | -23.0 | Yes |
|  |  |  |  |  |  |  | ASBVdB-sRNA39a | 21 | 2.0 | 2.0 | -23.0 | Yes |
|  |  |  |  |  |  |  | ASBVdB-sRNA39b | 21 | 2.0 | 2.0 | -23.0 | Yes |
|  |  |  |  |  |  |  | ASBVdB-sRNA39c | 21 | 2.0 | 2.0 | -23.1 | Yes |
|  |  |  |  |  |  |  | ASBVdB-sRNA38a | 20 | 3.0 | 3.0 | -22.1 | Yes |
|  |  |  |  |  |  |  | ASBVdB-sRNA41a | 19 | 3.0 | 3.0 | -22.1 | Yes |
| C11g021230 | Pentatricopeptide repeat-containing protein At1g10270 | -0.60 | 0.01 | -0.52 | 0.01 | Mitochondrion | ASBVdB-sRNA3 | 24 | 3.0 | 3.0 | -33.2 | No |
|  |  |  |  |  |  |  | ASBVdB-sRNA7a | 25 | 3.0 | 3.0 | -31.6 | No |
|  |  |  |  |  |  |  | ASBVdB-sRNA1 | 25 | 3.0 | 3.0 | -33.5 | No |
|  |  |  |  |  |  |  | ASBVdB-sRNA8 | 24 | 3.0 | 3.0 | -31.6 | No |
|  |  |  |  |  |  |  | ASBVdB-sRNA7b | 25 | 3.0 | 3.0 | -31.6 | No |
| C06g015900 | E3 ubiquitin-protein ligase arkadia-A, putative | -1.33 | 0.01 | -1.92 | 1.007E-03 | Cytoplasm /Nucleus | ASBVdB-sRNA23 | 20 | 3.0 | 4.0 | 0.0 | Yes |
|  |  |  |  |  |  |  | ASBVdB-sRNA39a | 21 | 3.0 | 4.0 | 0.0 | Yes |
|  |  |  |  |  |  |  | ASBVdB-sRNA39b | 21 | 3.0 | 4.0 | 0.0 | Yes |
|  |  |  |  |  |  |  | ASBVdB-sRNA39c | 21 | 3.0 | 4.0 | 0.0 | Yes |
|  |  |  |  |  |  |  | ASBVdB-sRNA38a | 20 | 3.0 | 4.0 | 0.0 | Yes |
|  |  |  |  |  |  |  | ASBVdB-sRNA35 | 22 | 3.0 | 4.0 | 0.0 | Yes |
|  |  |  |  |  |  |  | ASBVdB-sRNA41a | 19 | 3.0 | 4.0 | 0.0 | Yes |
|  |  |  |  |  |  |  | ASBVdB-sRNA34 | 21 | 3.5 | 4.5 | 0.0 | Yes |
|  |  |  |  |  |  |  | ASBVdB-sRNA32 | 22 | 3.5 | 4.5 | 0.0 | Yes |
|  |  |  |  |  |  |  | ASBVdB-sRNA37 | 19 | 3.5 | 4.5 | 0.0 | Yes |
| C02g035850 | 40S ribosomal protein S15a | -0.51 | 0.02 | -0.56 | 0.02 | Cytoplasm | ASBVdB-sRNA15 | 23 | 3.0 | 3.5 | 0.0 | Yes - with G:U |
| C04g000140 | Aspartate--tRNA ligase | -0.90 | 2.418E-03 | -0.88 | 3.014E-03 | Cytoplasm | ASBVdB-sRNA15 | 23 | 3.0 | 3.5 | 0.0 | Yes - with G:U |
| C03g048730 | serine/threonine-protein kinase pakF-like | -1.37 | 0.11 | -1.29 | 0.07 | Nucleus | ASBVdB-sRNA24 | 25 | 3.0 | 3.0 | -25.4 | Yes |
|  |  |  |  |  |  |  | ASBVdB-sRNA29 | 23 | 3.5 | 4.0 | 0.0 | Yes |
|  |  |  |  |  |  |  | ASBVdB-sRNA26b | 25 | 3.5 | 4.0 | 0.0 | Yes |
| C02g040700 | Unknown protein | -1.04 | 2.193E-03 | -0.90 | 0.02 | Cytoplasm/Plastid | ASBVdB-sRNA7a | 25 | 3.5 | 3.5 | -29.2 | No |
|  |  |  |  |  |  |  | ASBVdB-sRNA8 | 24 | 3.5 | 3.5 | -29.2 | No |
|  |  |  |  |  |  |  | ASBVdB-sRNA7b | 25 | 3.5 | 3.5 | -29.2 | No |
| C01g051500 | Peroxidase superfamily protein | -1.64 | 9.870E-04 | -1.80 | 4.240E-04 | Nucleus | ASBVdB-sRNA38a | 20 | 3.5 | 4.0 | 0.0 | Yes - with mismatch |
|  |  |  |  |  |  |  | ASBVdB-sRNA35 | 22 | 3.5 | 4.0 | 0.0 | Yes - with mismatch |
|  |  |  |  |  |  |  | ASBVdB-sRNA41a | 19 | 3.5 | 4.0 | 0.0 | Yes - with mismatch |
| C09g014610 | ATP citrate synthase | -0.70 | 2.340E-03 | -1.00 | 1.156E-04 | Cytoplasm | ASBVdB-sRNA25 | 24 | 3.5 | 4.0 | 0.0 | Yes |
| C10g012530 | Pentatricopeptide repeat-containing protein At2g20540 | -0.91 | 6.134E-04 | -0.89 | 1.072E-03 | Nucleus | ASBVdB-sRNA15 | 23 | 3.5 | 4.0 | 0.0 | Yes |
| C12g004290 | Signal recognition particle 9 kDa protein | -0.37 | 0.11 | -0.58 | 0.11 | Cytoplasm /Nucleus | ASBVdB-sRNA15 | 23 | 3.5 | 4.0 | 0.0 | Yes |
| C01g017850 | Protein GrpE | -1.08 | 2.973E-08 | -1.55 | 1.429E-13 | Plastid | ASBVdB-sRNA41b | 19 | 3.5 | 4.0 | 0.0 | Yes |
| C07g004620 | Xyloglucan endotransglucosylase /hydrolase | -1.32 | 4.95E-03 | -1.19 | 3.52E-03 | Extracellular | ASBVdB-sRNA41b | 19 | 3.5 | 3.5 | -18.3 | Yes |

^a^Target gene identifier and putative annotation in the *Persea americana* West Indian pure accession genome (Peame105).

^b^Log_2_(Fold Change) (log_2_FC) and adjusted *p*-value (*p*adj) of target gene expression in yellow sectors (SY) relative to green sectors (SG) of bleached leaves.

^c^Log_2_FC and *p*adj of target gene expression in yellow sectors of bleached leaves (SY) relative to asymptomatic (fully green) leaves (AS).

^d^Subcellular localisation of target protein as predicted by DeepLoc 2.1 software (https://services.healthtech.dtu.dk/services/DeepLoc-2.1/).

^e^E-value score for ASBVd-sRNA:mRNA target duplex from psRNATarget (https://www.zhaolab.org/psRNATarget/analysis) prediction at default parameters.

^f^E-value score for ASBVd-sRNA:mRNA target duplex from psRNATarget prediction at stricter parameters recommended by Fahlgren and Carrington (2010) (with penalties doubled in seed region).

^g^Minimum free energy (mfe) of ASBVd-sRNA:mRNA target duplex as predicted by RNAhybrid (https://bibiserv.cebitec.uni-bielefeld.de/rnahybrid).

^h^Presence of the full region containing the pathogenic determinant in the duplex formed between ASBVd-sRNAs and mRNA targets. Cases where the pathogenic region was interrupted by mismatches, bulges or G:U base pairing within the duplex are indicated.
